# Targeting sphingosine kinase 1 (SK1) enhances oncogene-induced senescence through ceramide synthase 2 (CerS2)-mediated generation of very-long-chain ceramides

**DOI:** 10.1038/s41419-020-03281-4

**Published:** 2021-01-04

**Authors:** Magali Trayssac, Christopher J. Clarke, Jeffrey L. Stith, Justin M. Snider, Naomi Newen, Christopher R. Gault, Yusuf A. Hannun, Lina M. Obeid

**Affiliations:** 1 Department of Medicine, Stony Brook University, Stony Brook, NY USA; 2 Stony Brook Cancer Center, Stony Brook, NY USA; 3 Virginia Mason Medical Center, Seattle, WA USA; 4 Northport Veterans Affairs Medical Center, Northport, NY USA

**Keywords:** Cancer metabolism, Senescence

## Abstract

Senescence is an antiproliferative mechanism that can suppress tumor development and can be induced by oncogenes such as genes of the Ras family. Although studies have implicated bioactive sphingolipids (SL) in senescence, the specific mechanisms remain unclear. Here, using MCF10A mammary epithelial cells, we demonstrate that oncogenic K-Ras (Kirsten rat sarcoma viral oncogene homolog) is sufficient to induce cell transformation as well as cell senescence—as revealed by increases in the percentage of cells in the G1 phase of the cell cycle, p21^WAF1/Cip1/CDKN1A^ (p21) expression, and senescence-associated β-galactosidase activity (SA-β-gal). Furthermore, oncogenic K-Ras altered SL metabolism, with an increase of long-chain (LC) C18, C20 ceramides (Cer), and very-long-chain (VLC) C22:1, C24 Cer, and an increase of sphingosine kinase 1 (SK1) expression. Since Cer and sphingosine-1-phosphate have been shown to exert opposite effects on cellular senescence, we hypothesized that targeting SK1 could enhance oncogenic K-Ras-induced senescence. Indeed, SK1 downregulation or inhibition enhanced p21 expression and SA-β-gal in cells expressing oncogenic K-Ras and impeded cell growth. Moreover, SK1 knockdown further increased LC and VLC Cer species (C18, C20, C22:1, C24, C24:1, C26:1), especially the ones increased by oncogenic K-Ras. Fumonisin B1 (FB1), an inhibitor of ceramide synthases (CerS), reduced p21 expression induced by oncogenic K-Ras both with and without SK1 knockdown. Functionally, FB1 reversed the growth defect induced by oncogenic K-Ras, confirming the importance of Cer generation in the senescent phenotype. More specifically, downregulation of CerS2 by siRNA blocked the increase of VLC Cer (C24, C24:1, and C26:1) induced by SK1 knockdown and phenocopied the effects of FB1 on p21 expression. Taken together, these data show that targeting SK1 is a potential therapeutic strategy in cancer, enhancing oncogene-induced senescence through an increase of VLC Cer downstream of CerS2.

## Introduction

Senescence is a mechanism by which cells enter a stable cell cycle arrest. It was originally discovered by Hayflick and Moorhead in 1961^1^ and further characterized by several others. In addition to cell cycle arrest, it is now recognized to manifest features including secretion of cytokines and chemokines, also known as the senescence-associated secretory phenotype (SASP), macromolecular damage, dysregulation of metabolism, and epigenetic and genetic changes^2^. Biomarkers widely used to screen for senescence are the senescence-associated-ß-galactosidase activity (SA-ß-gal)^3^, reflecting increased lysosomal functions and lipofuscin, caused by protein and lipid alterations^4^. Increased expression of p21^WAF1/Cip1/CDKN1A^ (p21) and enlarged cell size are also commonly used additional markers of senescence. Many intrinsic and extrinsic inducers of senescence have been identified, including telomere dysfunction, oncogene activation, and chemotherapeutics. Since the discovery that removal of senescent cells delayed the onset of age-related diseases^5,6^, senescence has become a target of interest for therapeutic intervention^7,8^. In cancer, senescence is considered as a tumor suppressor mechanism^9,10^ and, indeed, senescent cells are present in premalignant lesions but not malignant tumors^11^. However, the role of senescence is more complex as it can have deleterious effects in enhancing tumor progression by exacerbating inflammation through the SASP^12–15^. Senolytics, compounds that drive specifically the death of senescent cells, are needed to further explore the role of senescence in aging and age-related diseases, particularly in cancer^16,17^.

The bioactive sphingolipids (SL) ceramide (Cer), and sphingosine-1-phosphate (S1P) mostly exert opposite functions in the regulation of cell fate with Cer being anti-growth and pro-death while S1P is pro-growth and pro-survival^18^. Initial studies reported increased Cer is associated with replicative senescence^19,20^ and found that exogenous C6-Cer induced senescence in human fibroblasts^21^. Mechanistically, the effects of Cer appeared to be through Rb dephosphorylation^22^ and p21-mediated inhibition of CDK2^23^. Subsequently, senescence induction by exogenous Cer was reported by many groups and across a variety of cell types^24,25^. In cancer cells, Cer was reported to enhance senescence induced by chemotherapeutic drugs in pancreatic^26^ and lung cancer cells^27^ while deletion of acid ceramidase increased long-chain (LC) Cer and induced senescence of melanoma cells^28^. In vivo, our laboratory showed that loss of SK1 enhanced Cer levels and protected p53 null mice from thymic lymphoma development by inducing senescence^29^. On the other hand, S1P was found to interact with human telomerase reverse transcriptase (hTERT) to promote telomere maintenance and cancer progression^30^. Thus, a better understanding of how SL enzymes regulate senescence may lead to specific interventions to prevent tumor progression.

The Ras family of GTPases, including K-Ras, N-Ras, and H-Ras are well-known oncogenes that are mutated in 30% of cancers^31^. Their oncogenicity is often due to point mutations of which G12V is one of the most common. Mutant K-Ras, in particular, is common in pancreatic (90%), colorectal (30–50%), and lung cancer (25%)^32^. While Ras mutations in breast cancer are less common (<10%)^32^, Ras signaling is hyperactive in 50% of breast tumors^33–36^ owing to heightened growth factor receptor signaling.

Our laboratory found that oncogenic K-Ras modulates SL metabolism in human embryonic cells, in part through effects on sphingosine kinase 1 (SK1)^37^. This prompted us to evaluate how dysregulation of SL metabolism contributes to oncogene-induced senescence. Here, we find that targeting SK1 increases Cer levels and enhances K-Ras-induced senescence, and we identify the Cer species and the ceramide synthase (CerS) isoform involved. Overall, this work provides new insights into the functional significance of SL in oncogene-induced senescence.

## Materials and methods

### Cell culture and treatments

MCF10A were purchased from ATCC (Manassas, VA, USA) and cultured as previously described^38^. Cells were maintained at 37 °C, 5% CO_2_ in a humidified atmosphere and passaged every 3–4 days. Cells were authenticated by ATCC using STR analysis and tested every other month for mycoplasma contamination (MycoAlert Mycoplasma detection kit, #LT07-318, Lonza, Basel, Switzerland). Mouse embryonic fibroblast (MEF) WT or SK1−/− were generated and maintained as previously described^37^. Inhibitors used were PF-543 (Sigma, Saint-Louis, MO, USA), SKI-II (Cayman Chemicals, Ann Arbor, MI, USA), Myriocin (Sigma), Fumonisin B1 (Enzo Life Sciences, Farmingdale, NY, USA). For siRNA, cells were transfected with 20 nM siRNA using Lipofectamine RNAiMAX (Invitrogen, Carlsbad, CA, USA) according to manufacturer instructions. The siRNAs used are listed in Supplemental Table 1.

### Plasmids

pENTR4-V5 (Addgene, Watertown, MA, USA, #17425), pLenti CMV/TO Puro Empty (Addgene #17482), and pLenti CMV/TO Puro DEST (Addgene #17293) were gifts from Eric Campeau & Paul Kaufman. pBabe-KRas-GV (Addgene #9052) was a gift from William Hahn. pCMV-VSV-G (Addgene #8454) and pCMV-dR8.2 dvpr (Addgene #8455) were gifts from Bob Weinberg. Wild-type (WT) and K-Ras-GD mutants were generated from pBabe-K-Ras by site-direct mutagenesis. Ras constructs were subcloned into pENTR4-V5 and recombined into pLenti CMV/TO Puro DEST by clonase reaction.

### Generation of stable cell lines

Lentiviruses were used to generate isogenic MCF10A cell lines overexpressing empty vector, K-Ras-WT, -G12V, or -G12D. Lentiviral particles were produced by transfecting HEK-293T cells (ATCC) with 2 μg each of VSV-G, dVPR, and target constructs using X-tremeGENE 9 (Roche, Basel, Switzerland). Viral containing media was harvested 72 h post transfection, filtered (0.45 μm polyvinylidene fluoride filter), and stored at −80 °C. MCF10A cells were infected at 70% confluency using 8 μg/ml polybrene (Millipore, Burlington, MA, USA) and selected with an antibiotic (2μg/ml Puromycin, InVivoGen, San Diego, CA, USA) for 8 days.

### SDS-PAGE and immunoblotting

Protein extracts were prepared in RIPA buffer and analyzed by SDS-PAGE and immunoblotting as described previously^39^. Protein concentration was determined by BCA assay (Pierce, Appleton, WI, USA). Primary antibodies used are listed in Supplemental Table 1.

### Counting viable cell number

Cells in 6-well plates (50 or 100 K/well) were placed into media with or without EGF as indicated (day 0). At indicated times, cells were trypsinized, and viable cell numbers counted using the trypan blue and Countess cell counter (Invitrogen). If necessary, cells were transfected with siRNA or treated with pharmacological inhibitors 24 h after plating.

### Colony formation in soft agar

Cells (10 K/well) were plated in 6-well plates in media containing 0.3% agar (214530, BD Difco, Franklin Lakes, NJ, USA) over a solidified lower layer of media containing 0.6% agar) and incubated for 2 weeks. Cells were pretreated with pharmacological inhibitors or siRNA prior to plating. In total 200 μl growth medium was added to wells every 3–4 days to avoid drying out. Colonies were stained overnight with nitrotetrazolium blue chloride (Sigma, N6876) solution (1 mg/ml, phosphate-buffered saline (PBS)) and visualized using the EVOS XL Core Imaging System (AMG, Bothell, WA, USA).

### BrdU/PI staining for cell cycle analysis

Cells were incubated with 10 μM BrdU (51-2420KC, BD Pharmingen) for 60 min. After washing and scraping in PBS, cells were fixed in ice-cold 70% ethanol for 30 min at 4 °C. DNA denaturation was performed for 30 min at room temperature in 2 N HCl solution containing 0.5% Triton. Cells were neutralized with 0.1 M Na_2_B_4_O_7_ pH 8.5 and resuspended in 200 μl of 1% bovine serum albumin/0.5% Tween of washing buffer. In total 20 μl of anti-BrdU antibody FITC-coupled (51-23614 L, BD Pharmingen) was added and incubated for 30 min in the dark. Cells were pelleted, washed, and resuspended in 400 μl of PI/RNase staining solution (#4087, Cell Signaling, Danvers, MA, USA) containing 0.2% Triton. Cells were kept in the dark before analysis with a FACSCalibur machine at the Flow Cytometry Core Research Facility of Stony Brook University.

### SA-β-gal assay

After treatment, cells were fixed and stained using a SA-β-gal staining kit (#9860, Cell Signaling) according to manufacturer protocol. Pictures were taken using EVOS XL Core Imaging System (AMG).

### Lipofuscin staining

After 5 days in culture, cells were washed twice with PBS before and after fixation using PFA 4% for 10 min. Sudan Black B (#199664, Sigma) at 0.7% in 70% ethanol was dissolved by stirring at 60 °C overnight. The solution was then filtered and added to the cells for 8 min. After a quick wash with 70% ethanol, water was added and kept during the observation using EVOS XL Core Imaging System (AMG).

### Lipid analysis

Briefly, cells were washed on ice (PBS) and scraped in 2 ml of cell extraction buffer (70% isopropanol:ethyl acetate 2:3). Extraction and analysis of SL were performed as previously described^40^ at the Lipidomics Shared Resource Core of Stony Brook University. For SK activity, 250 nM C17-Sph was added to cells for the last hour of incubation^41^.

### Reverse transcription-quantitative PCR

RNA was extracted from cells using the PureLink RNA Mini Kit (Ambion, Austin, TX, USA) according to manufacturer protocol. RNA concentration was determined by NanoDrop (TM 2000c, Thermo Fisher Scientific, Waltham, MA, USA) and 1 μg of total RNA was converted to cDNA using SuperScript III SuperMix (Invitrogen, 11752-050). After diluting cDNA 1/15 in molecular biology water (W4502, Sigma), real-time quantitative PCR was performed on the system Applied Biosystems^TM^ 7500 (Thermo Fisher Scientific). The reaction volume was 20 μl containing 5 μl of cDNA template, 1 μl of TaqMan assays 20×, 10 μl of iTaq and 4 μl of molecular biology water. TaqMan assays used are listed in Supplemental Table 1. Ct values obtained were used to calculate mean normalized expression relative to actin as a housekeeping gene.

### Statistical analysis

The data are presented as mean ± standard error of the mean and GraphPad Prism 8 software (San Diego, CA, USA) was used for statistical calculations, and a *p* < 0.05 was considered sufficient to reject the null hypothesis. For comparison of two groups an unpaired *t* test assuming equal variance was performed. For comparison of more than two groups, a one-way ANOVA was performed with an appropriate post-test. For analysis of two variables, a two-way ANOVA with appropriate post-test was performed. Details are present in the figure legends and supplemental figure legends.

## Results

### Mutant K-Ras induces cell transformation and oncogene-induced senescence

**Fig. 1:**
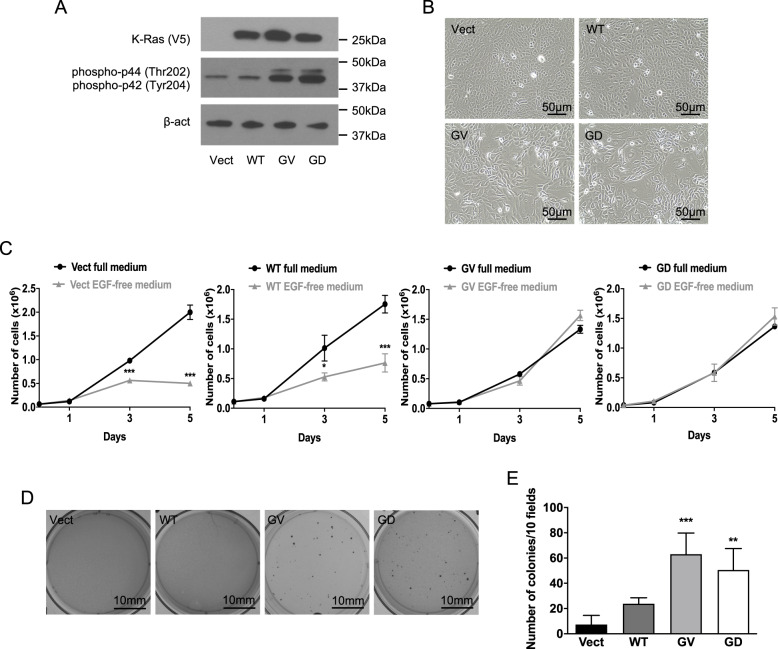
Mutant K-Ras overexpression induces cell transformation. **A** Western blot analysis of protein extracts from cells overexpressing Vector, WT, GV, or GD K-Ras cultured for 48 h. The overexpressed proteins WT K-Ras and mutant K-Ras GV and GD are V5-tagged. V5 tag, phosphoERK1/2, and ß-actin were used as primary antibodies. **B** Observation of morphology of cells overexpressing Vector, WT, GV, or GD K-Ras after 48 h in culture using the bright field. Cell morphology varies across different conditions. **C** Cell growth analysis using quantification of the cell number over the course of 5 days in culture. Cells overexpressing Vect, WT, GV, or GD K-Ras were plated in a growth medium that contains EGF. The next day, called day 0, the medium was changed to either growth medium (full medium, in black) or growth medium where EGF was not added (EGF-free medium, in gray). Cells were trypsinized and number of viable cells was determined using a hemocytometer after 1, 3, and 5 days (*N* = 4, data presented as mean ± SEM, two-way ANOVA with Sidak’s multiple comparisons test, * means *p* < 0.05 comparing the group full medium to the group EGF-free medium at the same time point). **D** Colony formation assay using cells overexpressing Vect, WT, GV, or GD K-Ras. Cells were plated and cultured in soft agar for 2 weeks and then finally stained overnight using a membrane-permeable dye that stains viable cells. **E** Quantification of the number of colonies obtained after culturing cells overexpressing Vect, WT, GV. or GD K-Ras for 2 weeks in soft agar (*N* = 4, data presented as mean ± SEM, one-way ANOVA with Dunnett’s multiple comparisons test, * means *p* < 0,05 comparing the tested groups to the group control Vector).

Because Ras signaling is hyperactive in a majority of breast cancers^33–36^ and oncogenic K-Ras was previously shown to induce senescence in fibroblasts^42–45^, intestinal^46^, and bronchial epithelial cells^45^, we decided to explore the role of SL in oncogene-induced senescence in breast epithelial cells. We stably transfected MCF10A—a non-transformed human cell line—with WT K-Ras or K-Ras-G12V or K-Ras-G12D, the two most clinically relevant mutants in human tumors^47^. Cells expressing empty vector were generated as controls. V5 immunoblots confirmed the expression of K-Ras constructs (Fig. 1A and Supplementary Fig. 1A) while analysis of downstream effectors of K-Ras^48^, showed that both mutants increased phospho-ERK1/2 levels (Fig. 1A and Supplementary Fig. 1B). Biologically, K-Ras mutants induced morphologic changes with cells appearing more fibroblastic and enlarged, consistent with transformation, in contrast to the classical cobblestone morphology of epithelial cells seen in vector and WT K-Ras cells (Fig. 1B). This was also observed in MEF using GV K-Ras mutant (Supplementary Fig. 2A). To confirm Ras-induced transformation, two well-established hallmarks of cell transformation were assessed: growth factor-independent and anchorage-independent growth. For the former, as MCF10A cells are highly dependent on exogenous EGF for proliferation, cell growth was analyzed in an EGF-free medium. EGF withdrawal significantly impeded the growth of vector and WT K-Ras cells but this had no effect on either mutant K-Ras GV or GD cells (Fig. 1C, in black, are the results obtained using full medium and in gray are those obtained with EGF-free medium). Studies on anchorage-independent growth showed that vector cells could not form colonies (<10 per 10 fields) whereas WT K-Ras induced a modest number of colonies (20 per 10 fields) (Fig. 1D, E). In contrast, mutant K-Ras cells showed a robust ability to form colonies (50–60 colonies per 10 fields) (Fig. 1D, E). Collectively, these results confirm that mutant K-Ras is able to transform MCF10A cells and MEF.

**Fig. 2:**
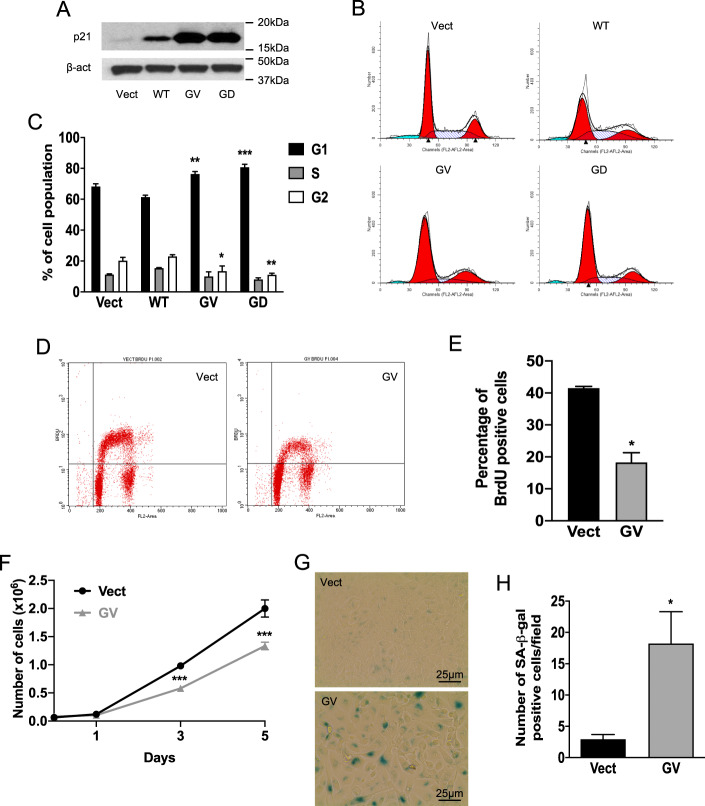
Oncogenic K-Ras overexpression induces cell senescence. **A** Western blot analysis of p21 expression in protein extracts from cells overexpressing Vect, WT, GV, or GD K-Ras after 48 h in culture. **B** Cell cycle analysis of cells overexpressing Vect, WT, or mutant K-Ras using propidium iodide staining (PI). Cells were plated and cultured for 48 h, then scraped and fixed and the cell pellet was resuspended in a solution of PI/RNase/Triton 15 min before flow cytometry analysis. **C** Quantification of cell cycle analysis to determine the percentage of cells in each phase of the cell cycle (*N* = 3, data are presented as mean ± SEM, two-way ANOVA with Dunnett’s multiple comparisons test, * means *p* < 0.05 comparing the tested groups to the group control Vector for one given phase). **D** Analysis of cell proliferation using BrdU incorporation. Cells were plated and kept in culture for 48 h and BrdU was added to the medium. Cells were then scraped and fixed. DNA was denatured and then cell pellets were incubated with an anti-BrdU antibody coupled to FITC. **E** Quantification of the percentage of cells BrdU positive (*N* = 3, data are presented as mean ± SEM, unpaired two-tailed *t* test, * means *p* < 0.05 comparing the tested group to the group control Vector). **F** Cell growth analysis performed by cell counting using cells that overexpress either Vect or GV K-Ras. A number of viable cells were measured after 1, 3, or 5 days in culture (*N* = 3, data are presented as mean ± SEM, two-way ANOVA with Sidak’s multiple comparisons test, * means *p* < 0.05 comparing the group tested to the group control Vector at the same time point). **G** SA-ß-gal assay in cells overexpressing Vector or GV K-Ras. Cells were plated and kept for 5 days in culture. They were then fixed and stained overnight using a SA-ß-gal staining kit. Positive cells are stained in blue. **H** Quantification of a number of SA-ß-gal positive cells (*N* = 5, data are presented as mean ± SEM, unpaired two-tailed *t* test, * means *p* < 0.05 comparing the tested group to the group control Vector).

Next, the effects of K-Ras on senescence in MCF10A cells were assessed. Immunoblot analysis of p21 revealed it was strongly increased by mutant K-Ras and modestly increased in WT K-Ras cells (Fig. 2A and Supplementary Fig. 1C). Comparable effects were seen with p27, another CDK inhibitor, and senescence marker (Supplementary Fig. 3A, B). Of note, we looked at the effects of K-Ras on the expression of other proteins involved in cell cycle regulation such as p53, Rb, cyclins, and CDK. No change was observed for p53 and Rb (Supplementary Figs. 3C–F) whereas mutant K-Ras induces a strong increase of Cyclin D1, Cyclin D2, CDK2, and CDK6 (Supplementary Figs. 3G–N). Importantly, cell cycle analysis showed that mutant K-Ras cells exhibited a higher percentage of cells in the G1 phase (80%) compared to Vect and WT K-Ras cells (<70%) (Fig. 2B, C) with a concomitant reduction in the G2 phase cells, suggesting mutant K-Ras induces a cell cycle arrest in the G1 phase. To confirm this, the incorporation of BrdU, a thymidine analog, was evaluated as a measure of cells cycling through the S phase. As can be seen, there was a significant reduction in BrdU-positive cells in K-Ras-GV cells (20%) compared to control cells (40%) (Fig. 2D, E), as would be expected with a G1 arrest. Further consistent with this, mutant K-Ras cells grew significantly less than Vector control cells after 3 and 5 days of culture (Fig. 2F). Finally, analysis of SA-ß-gal revealed strong staining in mutant K-Ras cells with little to no staining in control cells (Fig. 2G, H). Similarly, quantification of SA-ß-gal in MEF showed an increase of staining induced by GV K-Ras (Supplementary Fig. 2B). Furthermore, we performed Sudan Black B staining to detect lipofuscin, a more specific marker of senescent cells. Using replicative senescent fibroblasts as a positive control (Supplementary Fig. 4A), we observed that K-Ras GV cells show some staining whereas the control cells were all negative (Supplementary Fig. 4B). Of interest, some mutant K-Ras cells were strongly SA-β-gal positive while other cells were negative, suggesting the possibility of multiple cell populations. Nonetheless, taken together, these results show that oncogenic K-Ras induces cell senescence in MCF10A and MEF.

### Oncogenic K-Ras increases LC and very-LC Cer and SK1 protein levels

**Fig. 3:**
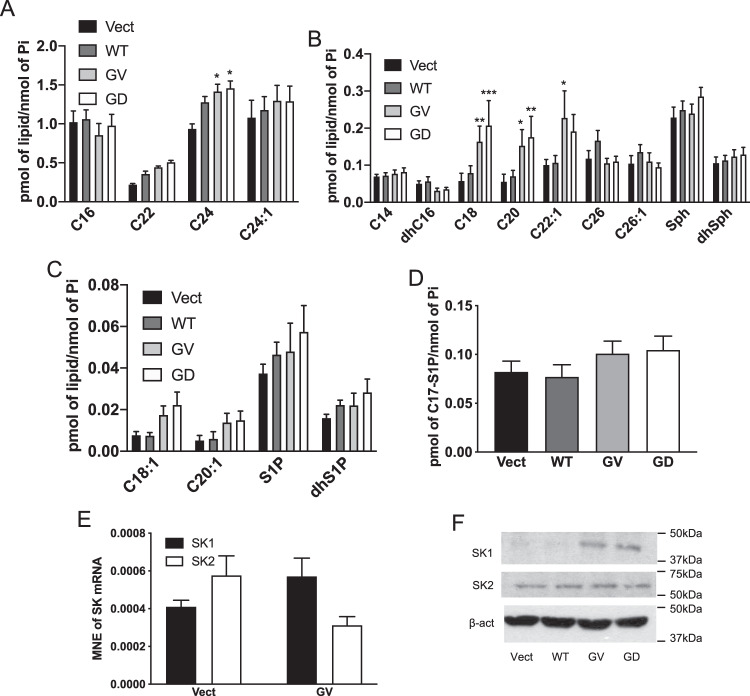
Oncogenic K-Ras overexpression increases long-chain and very-long-chain Cer and SK1 protein levels. **A**–**C** SL analysis using tandem LC/MS/MS in cells overexpressing Vect or WT or mutant K-Ras. Cells were plated for 48 h and then scraped in a lipid extraction buffer. SL levels were quantified and normalized to the amount of total lipid phosphate. SL was organized according to their amounts in the cells, from the most abundant in (**A**) to the less abundant in (**C**). Panel **B** shows the SL having intermediate levels in the cells (*N* = 6, data are presented as mean ± SEM, two-way ANOVA with Dunnett’s multiple comparisons test, * means *p* < 0.05 comparing the tested groups to the group control Vector). **D** Determination of SK activity using labeling with C17-Sph. Cells were plated and incubated for 48 h and then C17-Sph (250 nM) was added for 1 h and then the cells were scraped in the lipid extraction buffer and the samples were analyzed (*N* = 4, data are presented as mean ± SEM, one-way ANOVA with Dunnett’s multiple comparisons test, * means *p* < 0.05 comparing the tested groups to the group control Vector). **E** Analysis of SK mRNA levels using RT-qPCR in cells overexpressing Vect or GV K-Ras. Cells were plated and kept in culture for 48 h then scraped and RNA extraction was performed on the cell pellets using a kit. Another kit was used to synthesize cDNA using 1 µg of RNA. cDNA was then diluted and used as a template for RT-qPCR. SK1, SK2, and Actin TaqMan probes were used. The mean normalized expression of SK1 and SK2 was determined by using the Ct values (*N* = 4, data are presented as mean ± SEM, two-way ANOVA with Tukey’s multiple comparisons test, * means *p* < 0,05 comparing all the groups). **F** Western blot analysis of SK1 and SK2 protein expression in cells overexpressing Vect or WT or mutant K-Ras after 48 h in culture.

Next, we evaluated if oncogenic K-Ras modulates SL in the MCF10A system. WT K-Ras had no significant effects on most SL species (Fig. 3). In contrast, both K-Ras mutants significantly increased C24 Cer, the most abundant SL, but had minimal effect on C16, C22, and C24:1 Cer (Fig. 3A). Analysis of less abundant Cer species showed both K-Ras GV and GD cells had elevated levels of C18 and C20 Cer (Fig. 3B), while C22:1 Cer was only increased in K-Ras GV cells. In addition, levels of Sph, dhSph, C14, dhC16, C26, and C26:1 Cer were unchanged by either mutant (Fig. 3B). Similarly, K-Ras mutant cells showed no differences in levels of C18:1, C20:1 Cer, S1P, or dhS1P compared to vector cells (Fig. 3C). In situ analysis found that K-Ras GV overexpression induced a modest, but not significant, increase of cellular SK activity (Fig. 3D) (as with our prior study^37^) without effects on expression of SK1 and SK2 at mRNA level (Fig. 3E). Strikingly, though, levels of SK1 protein but not SK2 were significantly increased in mutant K-Ras cells (Fig. 3F and Supplementary Fig. 1D, E). Overall, these results show that oncogenic K-Ras modulates SL metabolism in MCF10A, favoring an increase of LC C18, C20 Cer and very-LC C22:1, C24 Cer, and of SK1 protein levels.

### SK1 inhibition enhances senescence induced by oncogenic K-Ras

**Fig. 4:**
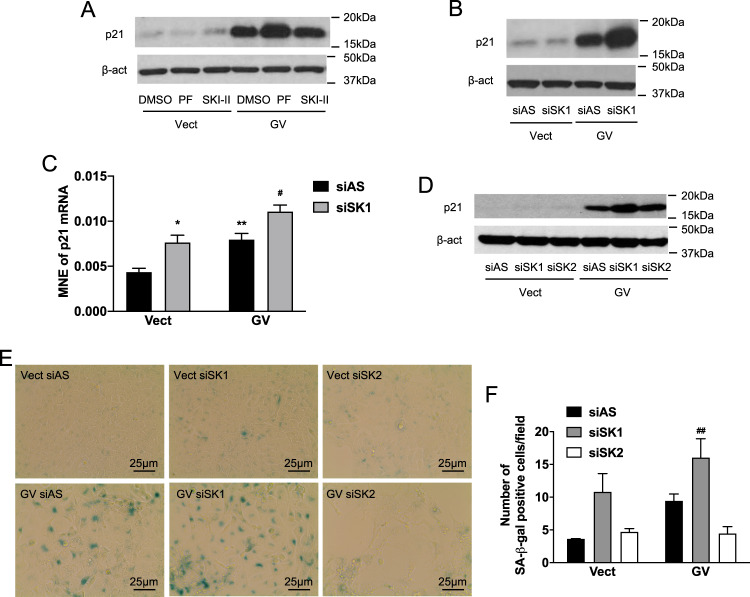
SK1 inhibition enhances senescence induced by oncogenic K-Ras overexpression. **A** Western blot analysis of p21 expression in cells overexpressing Vect or GV K-Ras. Cells were plated and treated the next day with pharmacological inhibitors PF-543 (100 nM) or SKI-II (10 μM) for 72 h. Proteins were extracted and p21 expression analyzed. **B** Western blot analysis of p21 expression in cell lysates from cells overexpressing Vect or GV K-Ras. Cells were plated and transfected the next day with siRNA control (AS) or SK1 (20 nM) for 72 h. Cells were then lysed and the protein level of p21 was determined. **C** Analysis of p21 expression by RT-qPCR in cells overexpressing Vect or GV K-Ras. Cells were plated and transfected the next day with control (AS) or SK1 siRNA (20 nM) for 72 h. Cells were then scraped and RNA extracted. cDNA was synthesized and used as a template for the RT-qPCR. Actin was used as a housekeeping gene. The mean normalized expression of p21 mRNA was determined by using the Ct values (*N* = 5, data are presented as mean ± SEM, two-way ANOVA with Tukey’s multiple comparisons test, * means *p* < 0.05 comparing the group tested to Vector control siRNA, ^#^ means *p* < 0.05 comparing the group tested to GV control siRNA). **D** Western blot analysis of p21 expression in protein lysates of cells overexpressing Vect or GV mutant K-Ras were treated for 72 h with siRNA control (AS) or directed against SK1 or SK2 (20 nM). **E** SA-ß-gal assay in cells overexpressing Vector or GV K-Ras. Cells were plated and transfected the next day with control (AS) or SK1 or SK2 siRNA (20 nM) and kept in culture for 5 days. They were then fixed and stained overnight using an SA-ß-gal staining kit. Positive cells are stained in blue. **F** Quantification of a number of SA-ß-gal positive cells 5 days after transfection with siRNA in cells overexpressing or not mutant K-Ras (*N* = 3, data are presented as mean ± SEM, two-way ANOVA with Tukey’s multiple comparisons test, * means *p* < 0.05 comparing the group Vector control siRNA to the group GV control siRNA, ^#^ means *p* < 0.05 comparing the group GV control siRNA to the group GV SK siRNA).

Based on the proposed roles of Cer and S1P in senescence, we evaluated the effect of targeting SK on oncogenic K-Ras-induced senescence. For this, although both mutants behaved similarly and manifested the same features, we chose to use K-Ras GV cells as they gave more consistent and robust effects. For a pharmacological approach, PF-543 (100 nM) and SKI-II (10 μM) were used as inhibitors of SK1 and SK2, respectively. The results showed robust effects of PF-543 but not SKI-II on SK activity (Supplementary Fig. 5A, B), suggesting SK1 is the major contributor to SK activity. Functionally, p21 levels in K-Ras-GV cells were significantly increased by PF-543 but not SKI-II (Fig. 4A and Supplementary Fig. 1F). Importantly, neither inhibitor affected p21 expression in control cells. In addition, knockdown of SK1 with siRNA (siSK1#1, simply called siSK1) reduced SK activity (Supplementary Figure5C), SK1 mRNA and protein (Supplementary Fig. 6A, C), and this led to increased p21 in K-Ras-GV cells but not control cells (Fig. 4B and Supplementary Fig. 1G). This effect was seen with a second SK1 siRNA, called siSK1#2 (Supplementary Fig. 7). Analysis of p21 expression showed that basal p21 mRNA expression was significantly higher in K-Ras-GV cells compared to control cells, and this was further increased by SK1 knockdown (Fig. 4C). Knockdown of SK2 (siRNA validated in Supplementary Fig. 6B) had no effect on p21 levels of K-Ras-GV cells (Fig. 4D and Supplementary Fig. 1H). Furthermore, SK1 knockdown in K-Ras-GV cells significantly increased SA-ß-gal compared to negative control siRNA while SK2 siRNA had no significant effect (Fig. 4E, F). SK1 knockdown had modest effects in the control cells. In MEF, the lack of SK1 prevented the morphological changes induced by oncogenic K-Ras (Supplementary Fig. 2A) and enhanced the positivity for SA-ß-gal (Supplementary Fig. 2B). Taken together, these results demonstrate that loss of SK1 activity, but not SK2, enhances the induction of senescence in the context of oncogenic K-Ras.

Of note, we decided to focus on p21 to evaluate senescence in our study. Given that p21 is known to have oncogenic properties when its expression is independent of p53^49^, we interrogated the relationship between p21 and p53. We observed that p21 expression, both basally and under induced conditions, was p53-dependent (Supplementary Fig. 8A, B). This confirmed that p21 was a suitable read-out to pursue our objectives.

### SK1 knockdown does not affect major components of the SASP induced by oncogenic K-Ras

**Fig. 5:**
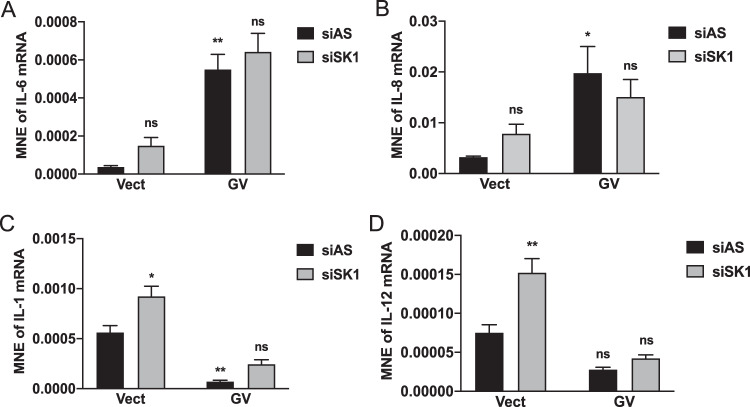
SK1 knockdown does not affect major components of the senescence-associated secretory phenotype induced by oncogenic K-Ras overexpression. **A**–**D** Analysis of expression of cytokines and chemokines at mRNA levels by RT-qPCR in cells overexpressing Vect or GV K-Ras that have been transfected with control (AS) or SK1 siRNA (20 nM) for 72 h. TaqMan probes for IL-6, IL-8, IL-1, and IL-12 were used and Actin was used as a control gene. The mean normalized expression of each mRNA was determined by using the Ct values (*N* = 3, data are presented as mean ± SEM, two-way ANOVA with Tukey’s multiple comparisons test, * means *p* < 0.05 comparing the group tested to Vector control siRNA, ^#^ means *p* < 0.05 comparing the group tested to GV control siRNA).

Senescent cells secrete many inflammatory mediators, well-known as the SASP^50^. Given the interest in targeting SK1 for cancer therapeutics^51–53^, we evaluated targeting SK1 on mRNA expression of SASP components (Fig. 5). For this, we analyzed the expression of IL-6 and IL-8—two established interleukins of the SASP. Mutant K-Ras significantly increased IL-6 and IL-8 levels compared to control cells, but surprisingly, SK1 knockdown had no effect on either (Fig. 5A, B). We also evaluated the protein level of IL-6 and found the same results (Supplementary Fig. 9A, B). We evaluated two other interleukins of interest: IL-1 can have pro-tumoral effects and can drive the expression of IL-6 and IL-8^54^. In contrast, IL-12 can induce tumor regression and promote senescence by regulating cytotoxic T and NK responses^55^. Results showed that K-Ras-GV decreased IL-1 expression while SK1 knockdown was able to enhance IL-1 mRNA in control cells but not K-Ras-GV cells (Fig. 5C). Similar experiments evaluating IL-1 protein levels weren’t conclusive since the protein was barely detectable (Supplementary Fig. 9C, D). K-Ras-GV had no significant effect on IL-12 expression, but SK1 knockdown increased IL-12 mRNA in control cells but not K-Ras-GV cells (Fig. 5D). Thus, although mutant K-Ras had distinct effects on various cytokines, SK1 knockdown does not augment these effects. This suggests that SK1 is uncoupled from the SASP in the context of oncogene-induced senescence.

### SK1 inhibition increases growth arrest induced by oncogenic K-Ras

**Fig. 6:**
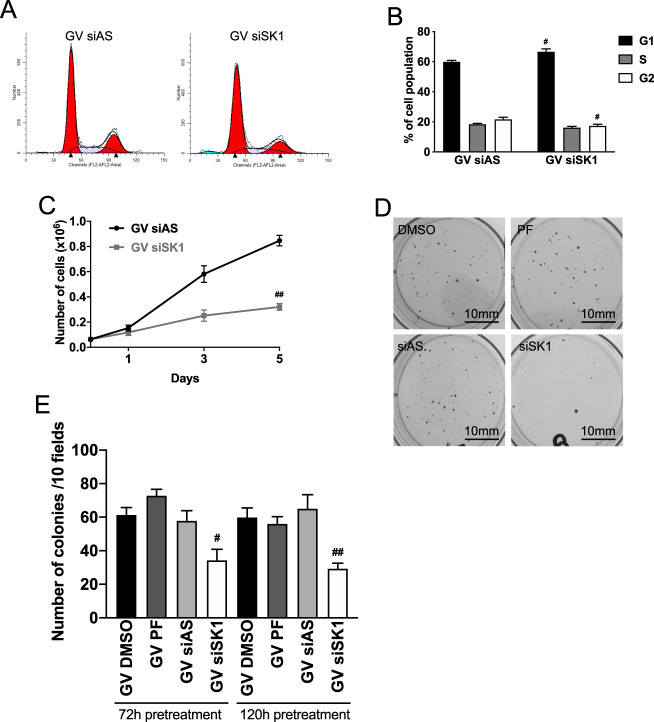
SK1 inhibition increases growth arrest induced by oncogenic K-Ras overexpression. **A** Cell cycle analysis of cells overexpressing mutant GV K-Ras using propidium iodide staining (PI). Cells were plated and transfected the next day with siRNA control (AS) or directed against SK1 (20 nM) for 72 h. Cells were then trypsinized and replated for 24 h before being scraped and fixed and resuspended in a solution of PI/RNase/Triton 15 min before flow cytometry analysis. **B** Quantification of cell cycle analysis to determine the percentage of cells in each phase of the cell cycle (*N* = 3, data are presented as mean ± SEM, two-way ANOVA with Dunnett’s multiple comparisons test, ^#^ means *p* < 0.05 comparing the tested groups to the group control GV for one given phase). **C** Cell growth analysis using quantification of the cell number over the course of 5 days in culture. Cells overexpressing GV K-Ras were plated. The next day, cells were transfected with control (AS) siRNA or SK1 siRNA (20 nM). Cells were trypsinized and the number of viable cells was determined using a hemocytometer after 1, 3, and 5 days (*N* = 5, data presented as mean ± SEM, two-way ANOVA with Sidak’s multiple comparisons test, ^#^ means *p* < 0.05 comparing the tested group to the group GV control siRNA at the same time point). **D** Colony formation assay using cells overexpressing GV K-Ras. Cells were plated and treated with PF-543 (100 nM) or transfected with control (AS) or SK1 siRNA (20 nM) for 72 h or 120 and then trypsinized and plated in soft agar for 2 weeks and then finally stained overnight using a membrane-permeable dye that stains viable cells. **E** Quantification of the number of colonies obtained after culturing cells overexpressing GV K-Ras for 2 weeks in soft agar. Cells were pretreated with either pharmacological inhibitor PF-543 or SK1 siRNA (*N* = 4, data are presented as mean ± SEM, one-way ANOVA with Dunnett’s multiple comparisons test, ^#^ means *p* < 0,05 comparing the group tested to GV control siRNA).

To determine the biological significance of the augmentation of senescence by loss of SK1, effects of SK1 loss on cell cycle, and cell growth in K-Ras-GV cells were assessed. Results showed that SK1 siRNA increased cells in the G1 phase with a concomitant decrease in G2 phase cells (Fig. 6A, B) leading to significant inhibition of cell growth (Fig. 6C). Importantly, the growth defect translated into a more complex model with SK1 knockdown leading to a significant decrease of colony formation in soft agar (Fig. 6D, E). Overall, these results show that SK1 downregulation strongly reduced anchorage-dependent and -independent growth in the context of oncogenic K-Ras. However, it should be noted that PF-543 (72 or 120 h) did not affect colony formation (Fig. 6D, E) possibly due to a lack of sustained inhibition of SK1. We obtained similar results with the MEF. SK1−/− MEF overexpressing oncogenic K-Ras grew less in anchorage-dependent (Supplementary Fig. 2C) and independent (Supplementary Fig. 2D) conditions compared to WT MEF overexpressing oncogenic K-Ras. This shows that downregulation or lack of SK1 leads to the same effect, an impaired ability of the cells to grow. It emphasizes a general role for SK1 in the regulation of oncogene-induced senescence.

### SK1 downregulation induces an increase of LC and very-LC Cer in oncogenic K-Ras cells

**Fig. 7:**
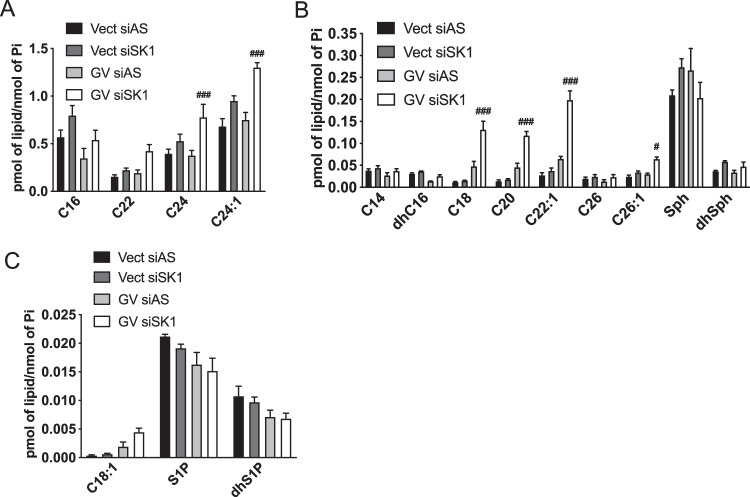
SK1 downregulation induces an increase of long-chain and very-long-chain Cer, in the context of oncogenic K-Ras overexpression. **A**–**C** SL analysis using tandem LC/MS/MS in cells overexpressing Vect or mutant GV K-Ras. Cells were plated and transfected the next day with control (AS) or SK1 siRNA (20 nM) for 72 h and then scraped in a lipid extraction buffer. SL levels were quantified and normalized to the amount of total lipid phosphate. SL was organized according to their amounts in the cells, from the most abundant in (**A**) to the less abundant in (**C**). Panel **B** shows the SL having intermediate levels in the cells (*N* = 4, data are presented as mean ± SEM, two-way ANOVA with Dunnett’s multiple comparisons test, ^#^ means *p* < 0.05 comparing the group GV SK1 siRNA to the group GV control siRNA).

The robust biological effects of SK1 downregulation in K-Ras-GV cells prompted us to investigate the SL that might be involved (Fig. 7). SK1 knockdown in K-Ras-GV cells significantly increased many Cer species including the more abundant C24 and C24:1 (Fig. 7A) and the lower abundance C18, C20, C22:1, C26:1 (Fig. 7B). Interestingly, loss of SK1 in control cells had no significant effects on lipid levels, not even a decrease in S1P (Fig. 7C). Overall, the strong effects of K-Ras and SK1 knockdown on LC and VLC C18-C26 Cer suggest they are the best candidates associated with the increased senescence.

### Ceramide synthases, especially CerS2, drive oncogenic K-Ras-induced senescence and mediate the increase of senescence induced by SK1 downregulation

**Fig. 8:**
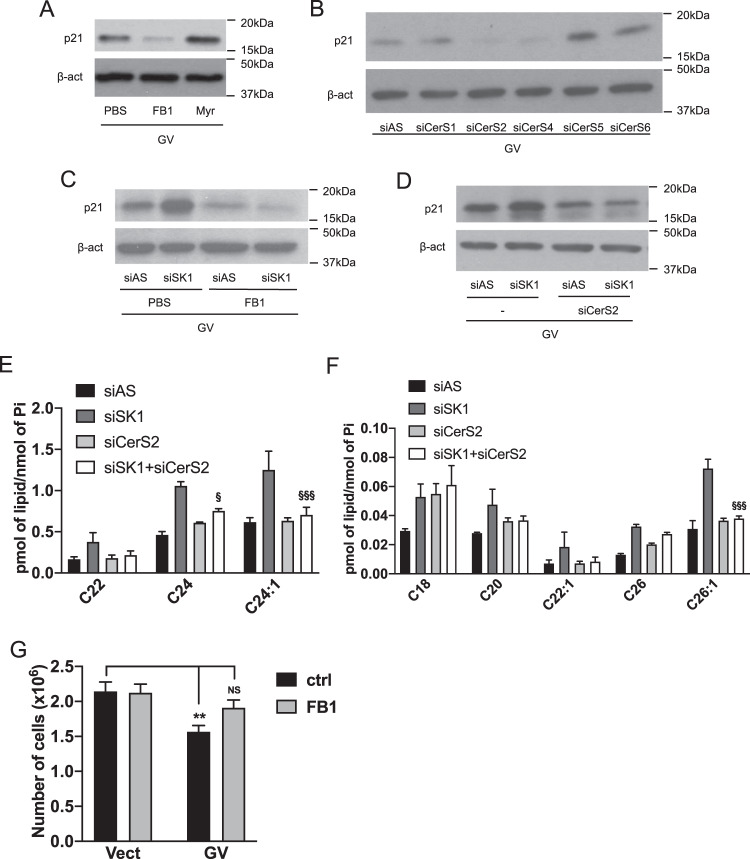
Ceramide synthases, especially CerS2, drive oncogenic K-Ras-induced senescence and mediate the increase of senescence induced by SK1 downregulation. **A** Western blot analysis of p21 expression in cell lysates from cells overexpressing GV K-Ras. Cells were plated and treated the next day with Fumonisin B1 (FB1) or Myriocin (Myr) for 72 h at 1 μM or 100 nM final, respectively. Cells were then lysed and the protein level of p21 was determined. **B** Western blot analysis of p21 expression in cell lysates from cells overexpressing GV K-Ras. Cells were plated and transfected the next day with control (AS) or CerS1-6 siRNA (20 nM) for 72 h. Cells were then lysed and the protein level of p21 was determined. **C** Western blot analysis of p21 expression in cell lysates from cells overexpressing GV K-Ras. Cells were plated and treated the next day with Fumonisin B1 (FB1) and transfected with control (AS) or SK1 siRNA (20 nM) for 72 h. Cells were then lysed and the protein level of p21 was determined. **D** Western blot analysis of p21 expression in cell lysates from cells overexpressing GV K-Ras. Cells were plated and cotransfected the next day with control (AS) or SK1 siRNA or control (AS) and CerS2 siRNA or SK1 and CerS2 siRNA (20 nM) for 72 h. Cells were then lysed and the protein level of p21 was determined. **E**, **F** SL analysis using tandem LC/MS/MS in cells overexpressing mutant GV K-Ras. Cells were plated and cotransfected the next day with control (AS) or SK1 siRNA or control (AS) and CerS2 siRNA or SK1 and CerS2 siRNA (20 nM) for 72 h and then scraped in a lipid extraction buffer. SL levels were quantified and normalized to the amount of total lipid phosphate (N = 3, data are presented as mean ± SEM, two-way ANOVA with Dunnett’s multiple comparisons tests, ^§^ means *p* < 0.05 comparing the group GV SK1 siRNA to the group GV SK1 + CerS2 siRNA). **G** Cell growth analysis of cells overexpressing mutant GV K-Ras. Cells were plated and treated the next day with FB1 (1 μM). A number of viable cells were evaluated after trypsinization every other day until 5 days in culture (*N* = 5, data are presented as mean ± SEM, two-way ANOVA with Tukey’s multiple comparisons test, * means *p* < 0,05 comparing the group tested to Vector control).

To determine the contribution of different classes of SL, we employed Fumonisin B1 (FB1; 1 μM) and Myriocin (Myr; 100 nM) as inhibitors of CerS and serine palmitoyltransferase, respectively. Results showed that FB1, but not myriocin, decreased p21 expression in mutant K-Ras cells (Fig. 8A and Supplementary Fig. 1I). Since FB1 inhibits all six CerS isoforms, siRNA against the different CerS were used (validation in Supplementary Fig. 6D–H). Overall the siRNAs were efficient except for CerS1 siRNA which did not suppress CerS1 mRNA levels in this cell line (and additional CerS1 siRNAs were ineffective). Therefore, we considered the result of this siRNA as an additional negative control. Expression analysis showed that CerS2 was the most expressed followed by CerS5, CerS6, CerS1, CerS4, and lastly CerS3 (Supplementary Fig. 10). Knockdown of CerS2 and CerS4 has the tendency to reduce in p21 expression in K-Ras-GV cells but this was not significant (Fig. 8B and Supplementary Fig. 1J). Having established basal effects of targeting CerS, it was important to assess their role in the context of SK1 loss. Importantly, both FB1 treatment (Fig. 8C and Supplementary Fig. 1K) and CerS2 siRNA (Fig. 8D and Supplementary Fig. 1L) abolished the increased p21 expression induced by SK1 siRNA in mutant K-Ras cells. To see if these changes are linked to specific Cer species, lipid levels were analyzed, specifically comparing differences between the effects of single SK1 and double SK1/CerS2 knockdown. As can be seen, LC Cer C18, C20, and some VLC Cer C22, C22:1, and C26 (Fig. 8E, F), were not significantly altered in the double transfection. In contrast, VLC Cer C24, C24:1, and C26:1 were increased by SK1 siRNA and this was reversed by double SK1/CerS2 knockdown siRNA. Finally, to link these findings to the previously observed biology, the effects of FB1 on cell growth were assessed (Fig. 8G). As can be seen, FB1 treatment effectively reversed the growth defect of K-Ras GV cells. Together with the results above, this suggests that VLC and not LC Cer drive the senescent phenotype. C24 Cer, specifically derived from CerS2, is potentially the key SL mediating oncogenic K-Ras-induced senescence and the additive effect of SK1 knockdown on this biology.

## Discussion

Given the interest in modulating SL metabolism for cancer therapy, it is perhaps surprising that the role of SL in oncogene-induced senescence has not been investigated. Here, we addressed this knowledge gap using oncogenic K-Ras and MCF10A breast epithelial cells as well as MEF. Results show that oncogenic K-Ras triggers transformation and senescence in MCF10A cells and in MEF. This is associated with increased LC and VLC Cer levels and increased SK1 protein. Biologically, targeting SK1 enhanced K-Ras-induced senescence in MCF10A cells and lack of SK1 in MEF gave similar results. Functionally, this translates into an impaired cell growth ability in both systems. In MCF10A, the enhancement of oncogene-induced senescence was associated with increases in LC and VLC Cer, without, to our surprise, affecting major components of the SASP. Importantly, targeting of CerS with FB1 and specific downregulation of CerS2 blunted the accumulation of VLC Cer induced by SK1 loss and reverted the senescent phenotype. Overall, this functionally links VLC Cer to K-Ras-induced senescence and suggests that targeting SK1 would be effective at suppressing tumor growth by enhancing senescence.

Our major results demonstrate a role for Cer in K-Ras-induced senescence and are most clearly evident from data showing that targeting SK1 leads to Cer accumulation and increased senescence in the presence of oncogenic K-Ras. A functional role for Cer is supported by FB1 treatment being effective at reverting biological effects induced by SK1 downregulation. This also suggests that Cer accumulation and not S1P loss is important for senescence induced by SK1 loss. These findings agree with prior studies reporting increased Cer in replicative senescent cells, and the capacity of exogenous Cer to induce senescence^20,21,56^. Mechanistically, our data implicate p21 as the primary downstream effector of Cer in regulating senescence, as also seen in pancreatic cancer cells and myoblasts^26,57^. Although Cer regulates multiple functions, growing evidence suggests individual Cer species can play distinct and opposing roles in the same biology. Prior studies into Cer and senescence have not investigated this owing to lack of MS technology at the time. Here, we connect LC and VLC Cer (C18–C26) to oncogene-induced senescence with specific loss of CerS2 reducing VLC Cer levels only, consistent with its known substrate specificity^58,59^. Notably, CerS2 loss enhances tumor number, and size in chemically induced hepatocellular carcinoma^60^. Our data here suggest that this could be due to alterations in senescence. Of note, S1P was reported to inhibit CerS2 activity^61^. Thus, a loss of SK1-derived S1P could activate CerS2, accounting for increased Cer levels. Alterations in CerS2 phosphorylation^62,63^ are also a possibility. These are currently being explored. Finally, we cannot fully exclude the role of CerS2-derived dhCer in the induction of senescence and this hypothesis is under investigation.

SK1 has been associated with anti-aging effects in different organs^64,65^, and indifferent organisms, e.g., *C. elegans*^66^. Here, linking Cer to oncogene-induced senescence and increased senescence observed following SK1 loss lead to the major conclusion that increased SK1 could be a mechanism to escape senescence. This also suggests that ectopic SK1 overexpression would be expected to overcome oncogene-induced senescence. We have attempted to investigate this but have had technical issues generating a double overexpressing oncogenic K-Ras and SK1 cell line and we acknowledge this as a limitation of our study. Nonetheless, this conclusion is supported by the higher SK1 levels in mutant K-Ras cells, similar to prior studies with H-RasG12V^67^. This extends our previous study connecting oncogenic K-Ras to SK1, and the current results suggest K-Ras regulates SK1 translation or protein stability. Of note, several micro RNA (miRNA) and long non-coding RNA (lncRNA) can negatively regulate SK1 translation^68^. Thus, oncogenic suppression of such miRNA and lncRNA could lead to increased SK1 levels.

In recent years, inducing senescence has emerged as a possible approach to restrain tumor growth and senolytics—agents that induce senescent cell death—have shown promise in clinical studies as potential therapies for aging^69^ and age-related diseases such as cancer^70^. However, in the context of cancer, the SASP has proved problematic. On the one hand, the SASP promotes local inflammation which could reinforce immunosurveillance and potentially clearance of the tumor^16^. Two-step treatment strategies inducing senescence as a primary event that is followed by immune clearance of the senescent cells have been efficient in several cancer cells and in preclinical models^71,72^. However, activation of the SASP may also trigger the proliferation and migration of malignant cells^73^. This could be circumvented by combining therapies with SASP inhibitors, which are currently under investigation in preclinical and clinical studies^74,75^. Alternatively, one could define targets that can induce senescence without impacting the SASP. In this context, our results establish SK1 as a viable target of interest as shown by the efficacy of SK1 downregulation at increasing oncogene-induced senescence but having no effect on major components of the SASP. This was somewhat surprising, as SK1 can regulate pro-inflammatory signaling in many systems including muscle^76^, adipose tissue^77^, and lung epithelial cells^78^. Conversely, SK1 was dispensable for IL-6 and IL-8 expression induced by lysophosphatidic acid in gastric cancer cells^79^. This suggests that the role of SK1 varies according to both cell type and stimulus but may need to be evaluated more thoroughly in the context of different oncogenic signals. Finally, while enhancing oncogene-induced senescence might be efficient, research into chemotherapy-induced senescence has suggested this might come at a long-term cost for patients. Studies have shown that tumor cells that escape from chemotherapy or radiation-induced senescence can acquire features of stemness^80–82^, and exhibit some similarities with cancer stem cells, thereby increasing their ability to drive tumor growth. Thus, to avoid such detrimental effects, targeting SK1 would need to be coupled with the removal of the senescent cells. Of note, these studies also suggest a strong heterogeneity of the senescent phenotype in cell populations and we are currently investigating some aspects of this concept.

In conclusion, our study establishes CerS2-derived VLC Cer as a mediator of senescence induced by K-Ras-GV through the regulation of p21 levels. Results further suggest that increase of SK1 is a mechanism by which transformed cells are able to escape the senescent phenotype. This supports the targeting of SK1 as a mean to induce oncogene-induced senescence, while potentially avoiding deleterious effects associated with activation of the SASP.

## Supplementary information

Revised Supplemental Figure 1

Revised Supplemental Figure 2

Revised Supplemental Figure 3

Revised Supplemental Figure 4

Revised Supplemental Figure 5

Revised Supplemental Figure 6

Revised Supplemental Figure 7

Revised Supplemental Figure 8

Revised Supplemental Figure 9

Revised Supplemental Figure 10

Revised Supplemental Table 1

Revised Supplemental Figure and Table legends
