# Supplementary figures and images for: Targeting sphingosine kinase 1 (SK1) enhances oncogene-induced senescence through ceramide synthase 2 (CerS2)-mediated generation of very-long-chain ceramides
Source: Cell Death Dis. 2021 Jan 4;12(1):27. doi: 10.1038/s41419-020-03281-4 (PMC7790826; doi:10.1038/s41419-020-03281-4)

## Slide 1
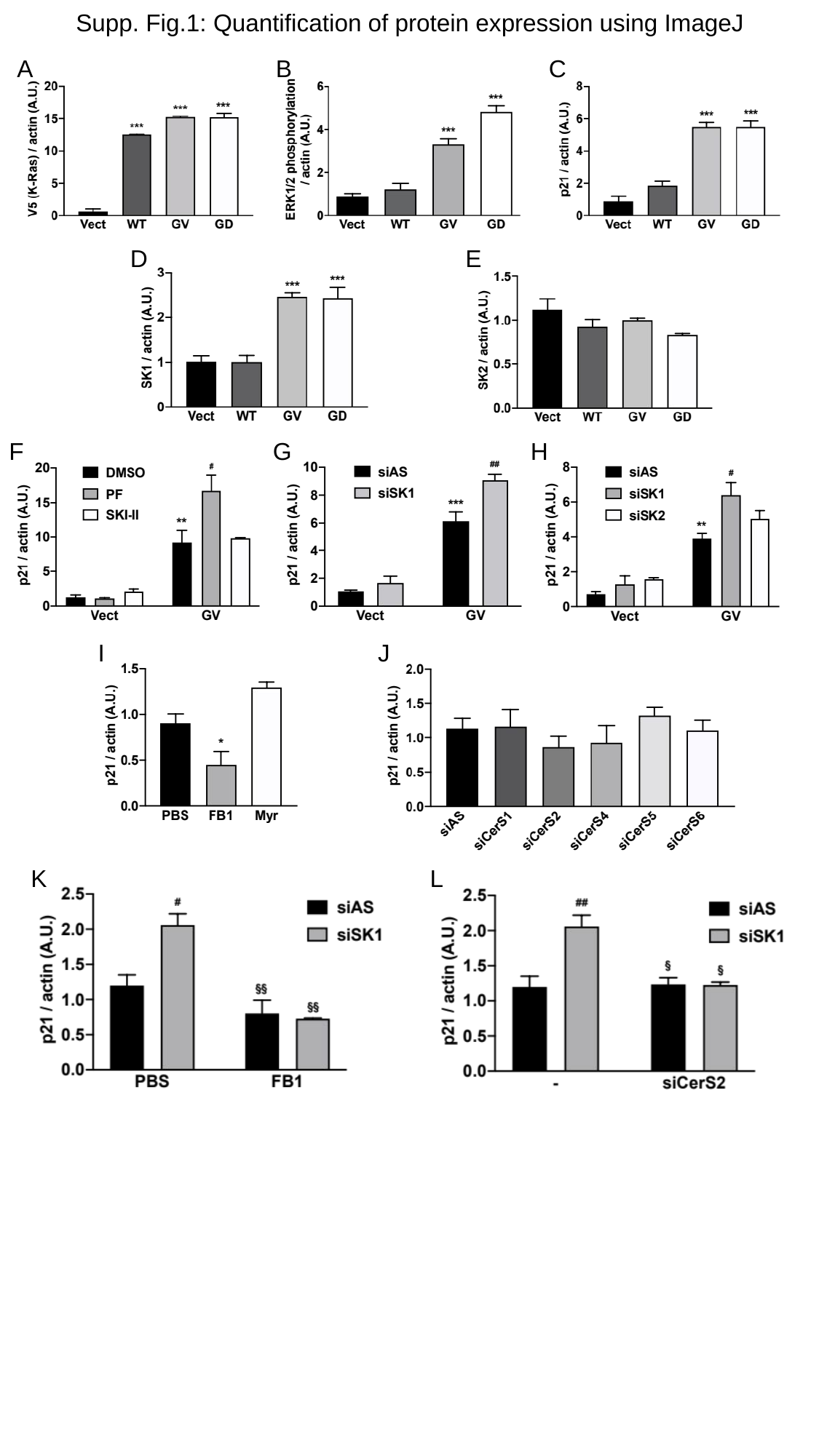

Supp. Fig.1: Quantification of protein expression using ImageJ
A
B
C
D
E
F
H
G
I
J
K
L

Supplement: Supplementary file 1 — Revised Supplemental Figure 1 [file 41419_2020_3281_MOESM1_ESM.pptx]

## Slide 1
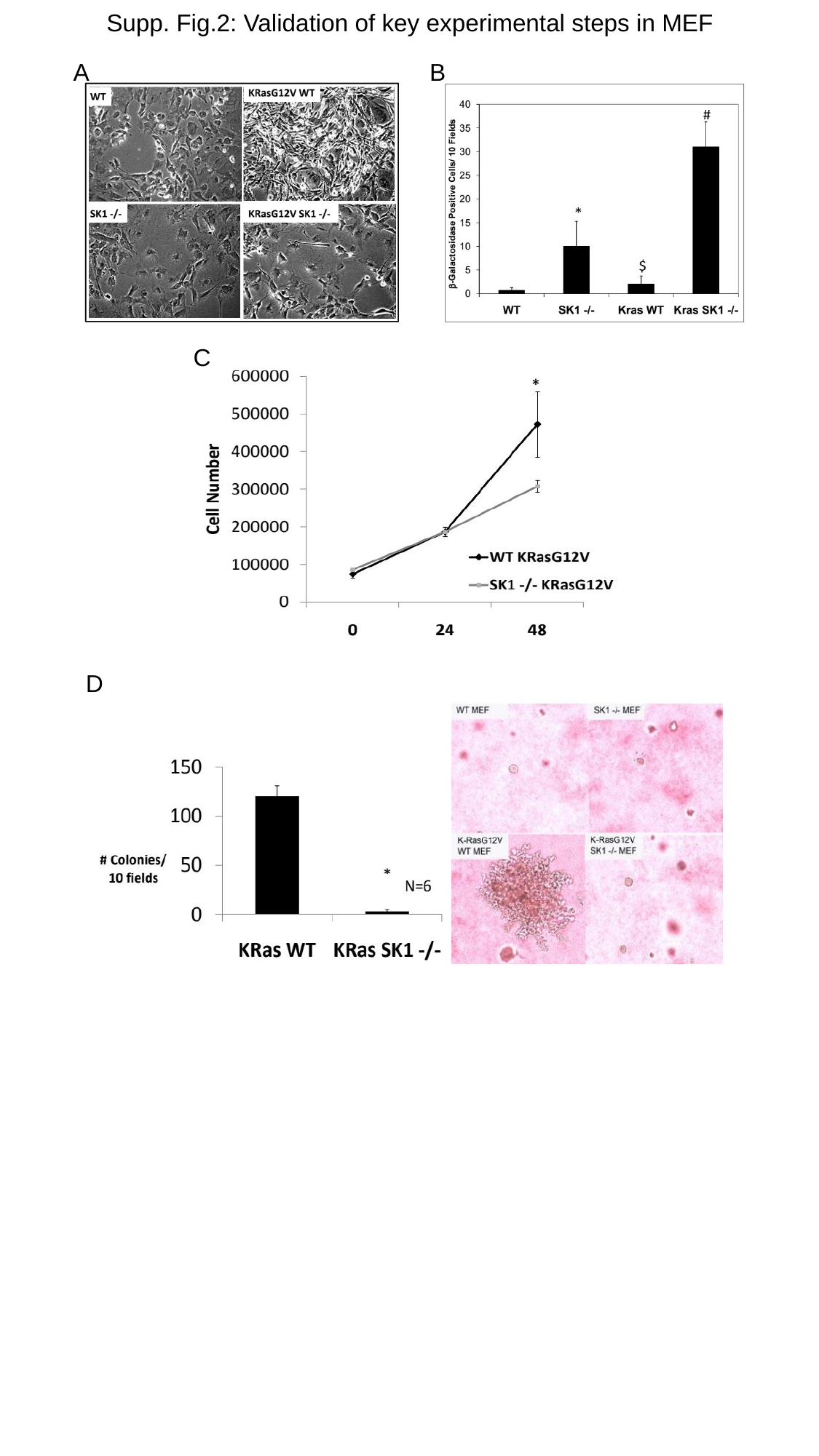

Supp. Fig.2: Validation of key experimental steps in MEF
A
B
C
D

Supplement: Supplementary file 2 — Revised Supplemental Figure 2 [file 41419_2020_3281_MOESM2_ESM.pptx]

## Slide 1
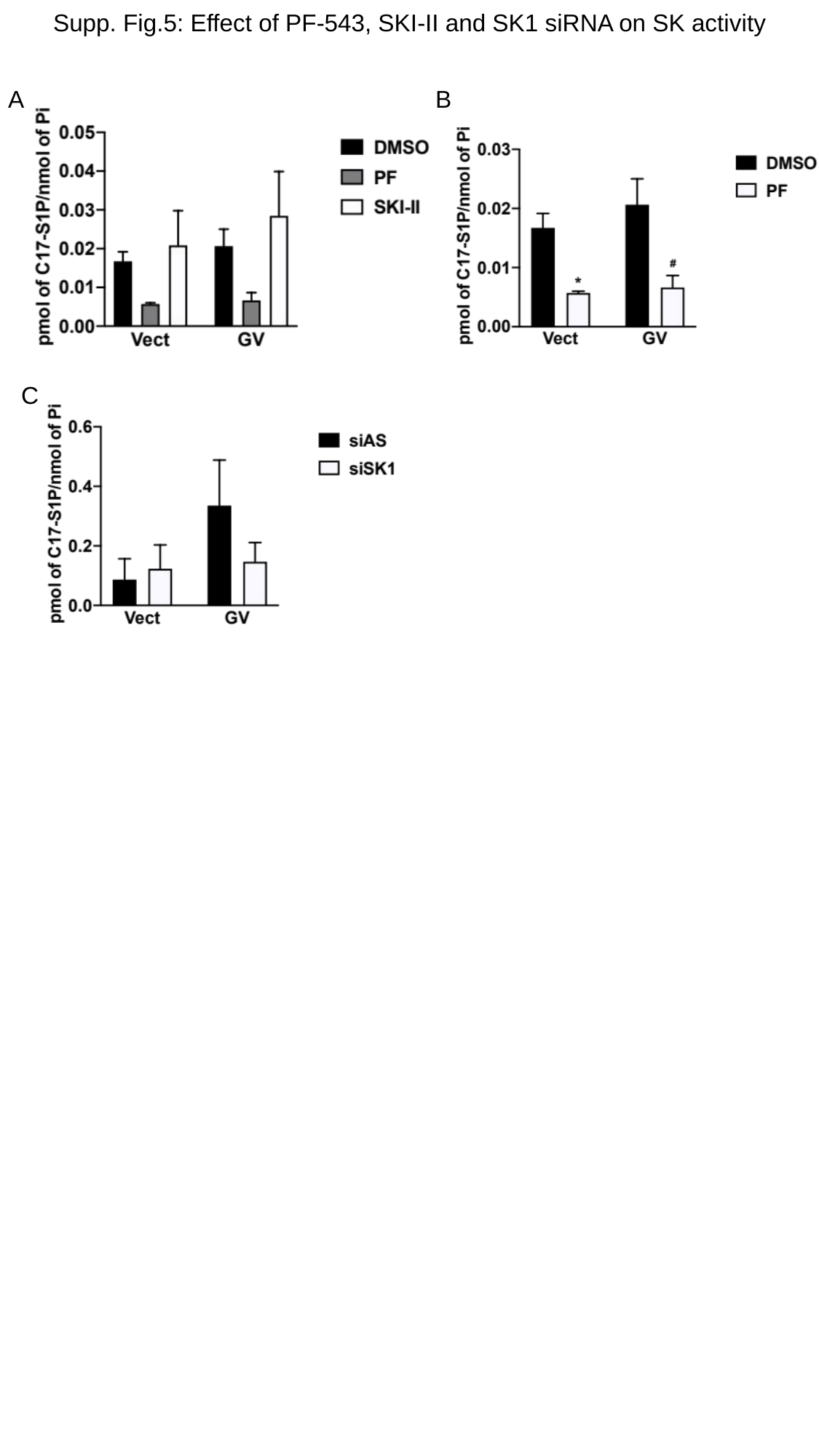

Supp. Fig.5: Effect of PF-543, SKI-II and SK1 siRNA on SK activity
A
B
C

Supplement: Supplementary file 5 — Revised Supplemental Figure 5 [file 41419_2020_3281_MOESM5_ESM.pptx]

## Slide 1
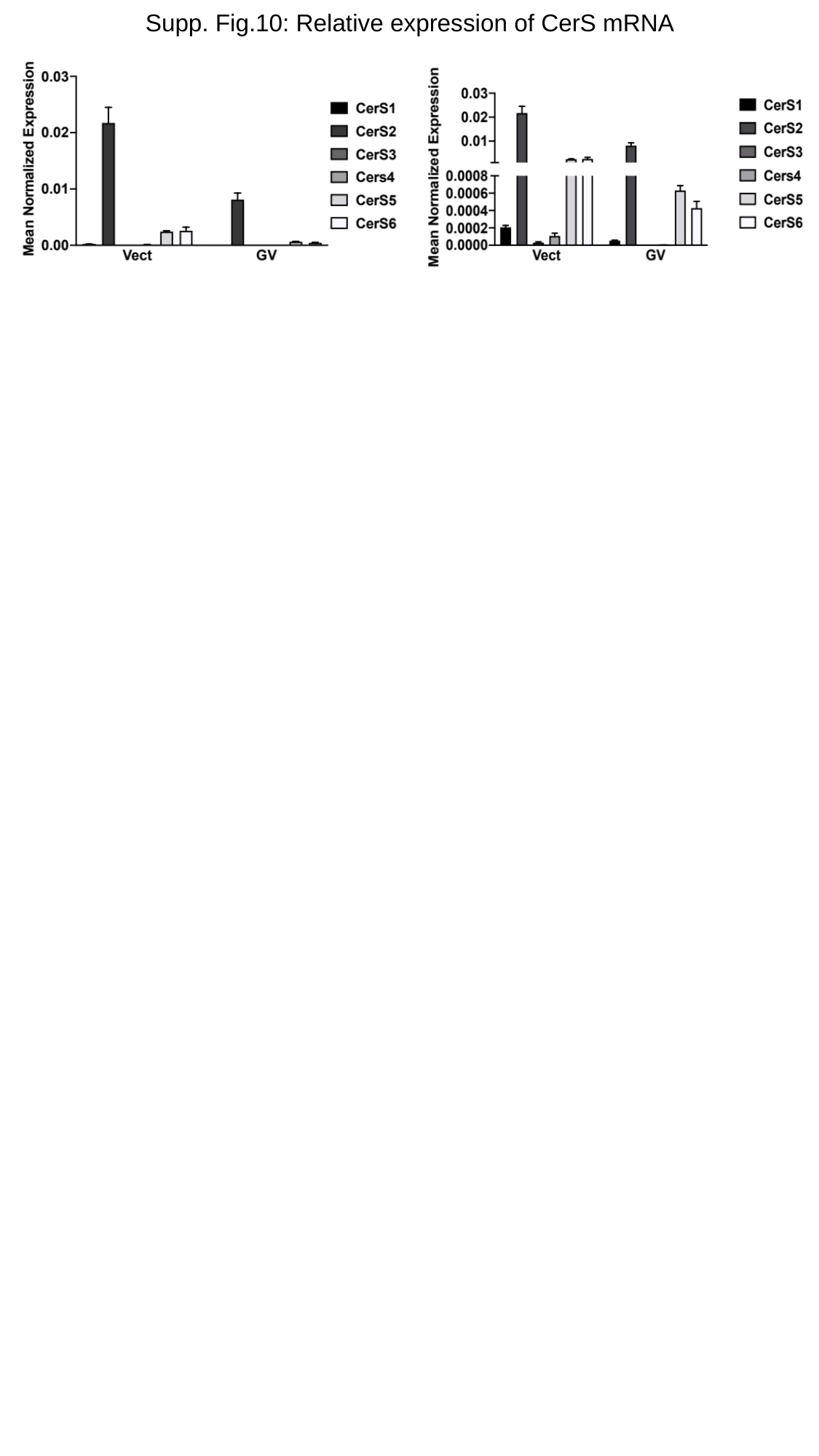

Supp. Fig.10: Relative expression of CerS mRNA

Supplement: Supplementary file 10 — Revised Supplemental Figure 10 [file 41419_2020_3281_MOESM10_ESM.pptx]

## Slide 1
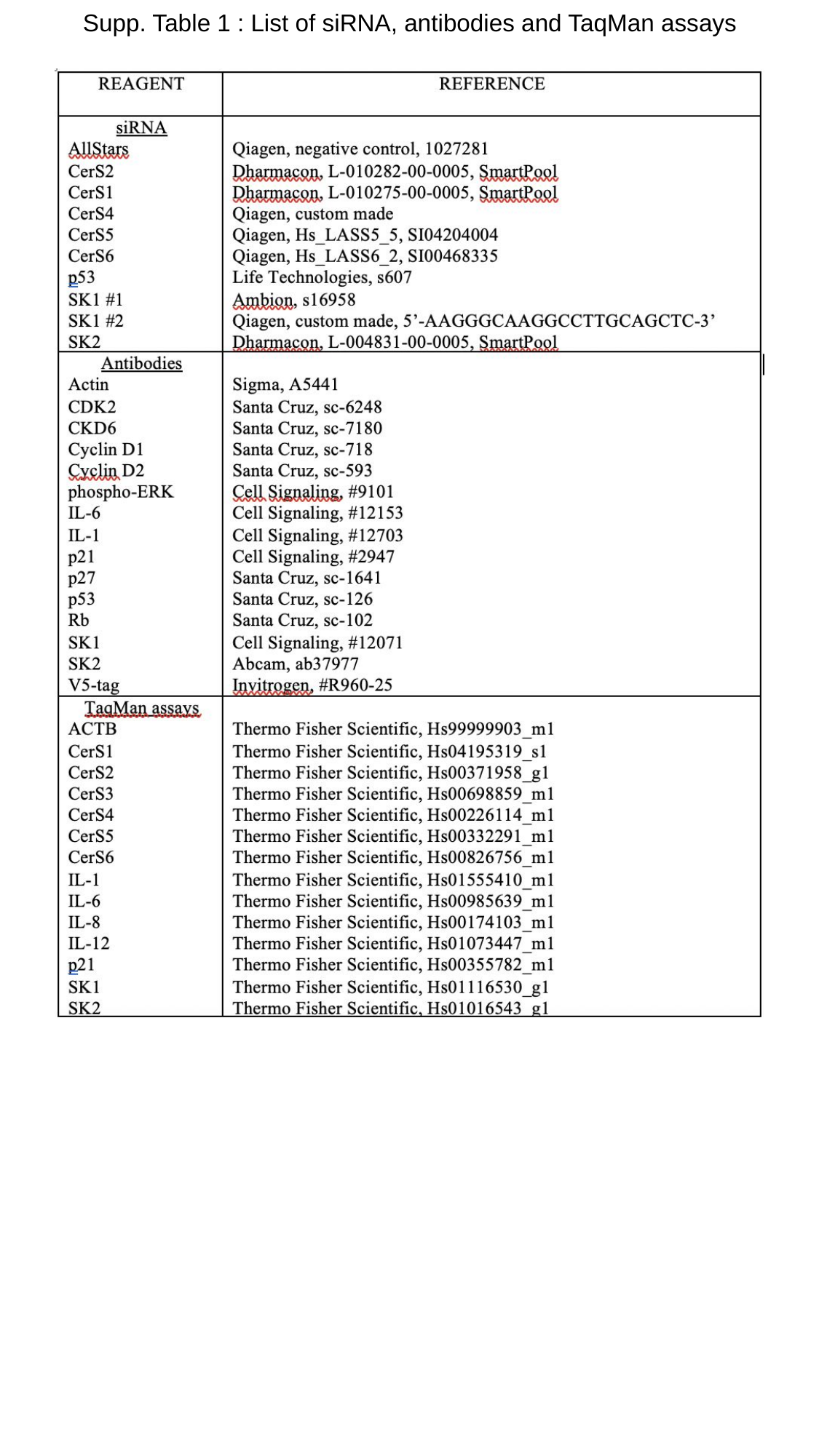

Supp. Table 1 : List of siRNA, antibodies and TaqMan assays

Supplement: Supplementary file 11 — Revised Supplemental Table 1 [file 41419_2020_3281_MOESM11_ESM.pptx]
