# Supplementary material for: Targeting sphingosine kinase 1 (SK1) enhances oncogene-induced senescence through ceramide synthase 2 (CerS2)-mediated generation of very-long-chain ceramides
Source: Cell Death Dis. 2021 Jan 4;12(1):27. doi: 10.1038/s41419-020-03281-4 (PMC7790826; doi:10.1038/s41419-020-03281-4)
Supplement: Supplementary file 3 — Revised Supplemental Figure 3 [file 41419_2020_3281_MOESM3_ESM.pptx]

## Slide 1
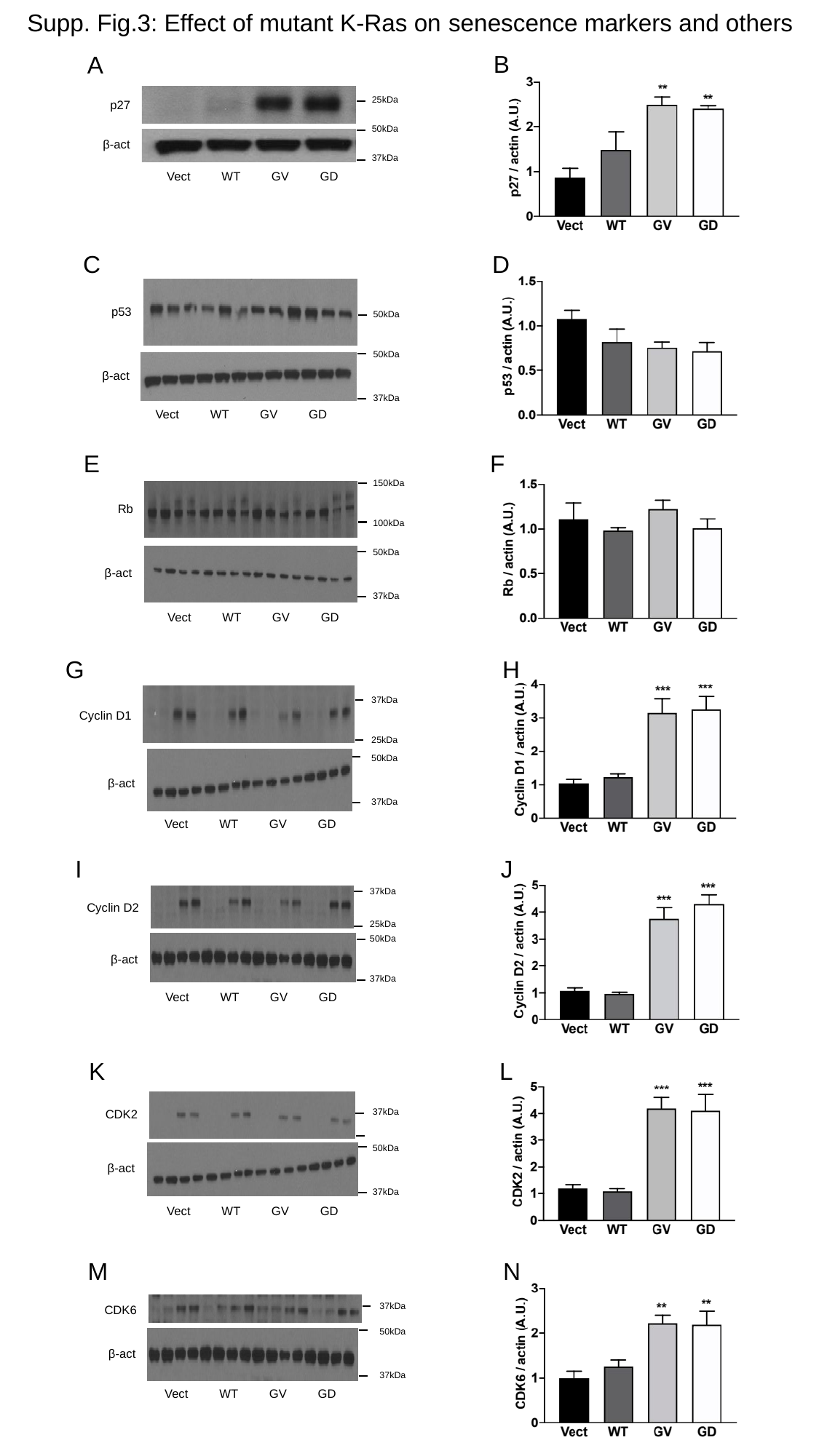

Supp. Fig.3: Effect of mutant K-Ras on senescence markers and others
B
A
25kDa
50kDa
37kDa
p27
β-act
Vect WT GV GD
C
D
p53
50kDa
50kDa
37kDa
β-act
Vect WT GV GD
E
F
150kDa
100kDa
50kDa
37kDa
Rb
β-act
Vect WT GV GD
H
G
37kDa
25kDa
50kDa
37kDa
Cyclin D1
β-act
Vect WT GV GD
I
J
37kDa
25kDa
50kDa
37kDa
Cyclin D2
β-act
Vect WT GV GD
K
L
37kDa
50kDa
37kDa
CDK2
β-act
Vect WT GV GD
M
N
37kDa
50kDa
37kDa
CDK6
β-act
Vect WT GV GD
