# Supplementary material for: Targeting sphingosine kinase 1 (SK1) enhances oncogene-induced senescence through ceramide synthase 2 (CerS2)-mediated generation of very-long-chain ceramides
Source: Cell Death Dis. 2021 Jan 4;12(1):27. doi: 10.1038/s41419-020-03281-4 (PMC7790826; doi:10.1038/s41419-020-03281-4)
Supplement: Supplementary file 4 — Revised Supplemental Figure 4 [file 41419_2020_3281_MOESM4_ESM.pptx]

## Slide 1
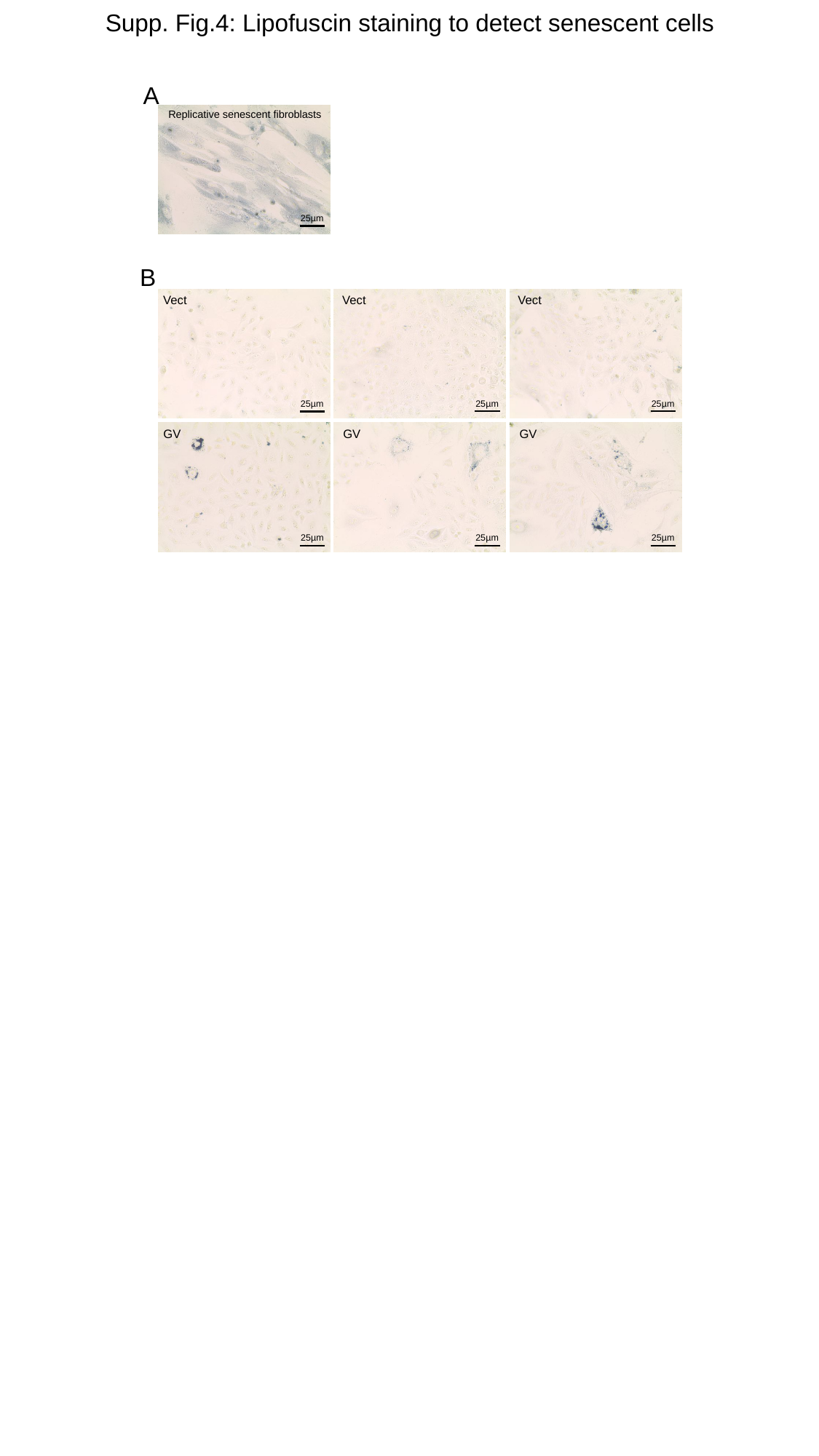

Supp. Fig.4: Lipofuscin staining to detect senescent cells
A
Replicative senescent fibroblasts
25µm
B
Vect Vect Vect
25µm
25µm
25µm
GV GV GV
25µm
25µm
25µm
