# Supplementary material for: Targeting sphingosine kinase 1 (SK1) enhances oncogene-induced senescence through ceramide synthase 2 (CerS2)-mediated generation of very-long-chain ceramides
Source: Cell Death Dis. 2021 Jan 4;12(1):27. doi: 10.1038/s41419-020-03281-4 (PMC7790826; doi:10.1038/s41419-020-03281-4)
Supplement: Supplementary file 6 — Revised Supplemental Figure 6 [file 41419_2020_3281_MOESM6_ESM.pptx]

## Slide 1
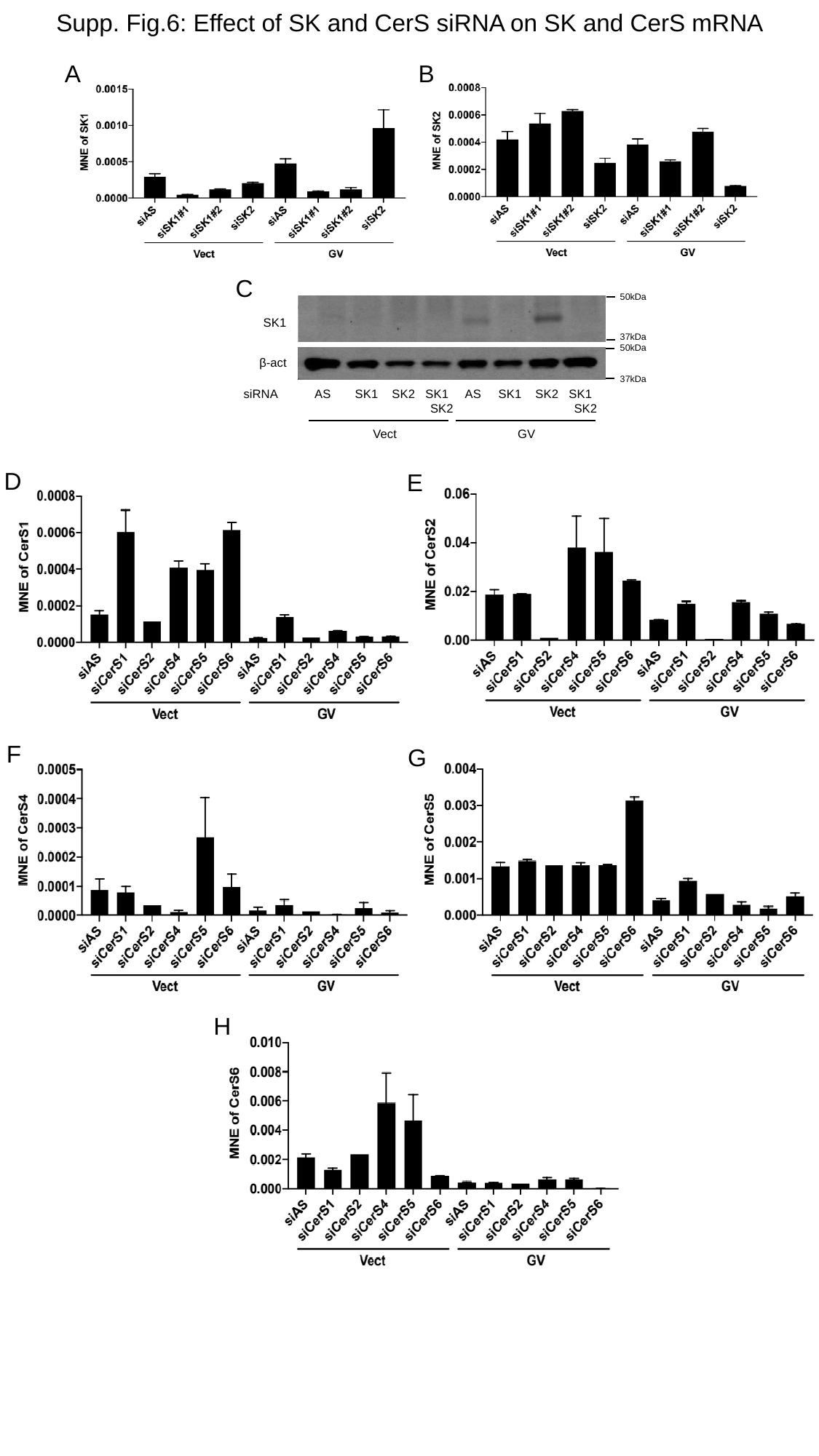

Supp. Fig.6: Effect of SK and CerS siRNA on SK and CerS mRNA
B
A
C
50kDa
37kDa
50kDa
37kDa
SK1
β-act
siRNA AS SK1 SK2 SK1 AS SK1 SK2 SK1
 SK2 SK2
 Vect GV
D
E
F
G
H
