# Supplementary material for: Targeting sphingosine kinase 1 (SK1) enhances oncogene-induced senescence through ceramide synthase 2 (CerS2)-mediated generation of very-long-chain ceramides
Source: Cell Death Dis. 2021 Jan 4;12(1):27. doi: 10.1038/s41419-020-03281-4 (PMC7790826; doi:10.1038/s41419-020-03281-4)
Supplement: Supplementary file 7 — Revised Supplemental Figure 7 [file 41419_2020_3281_MOESM7_ESM.pptx]

## Slide 1
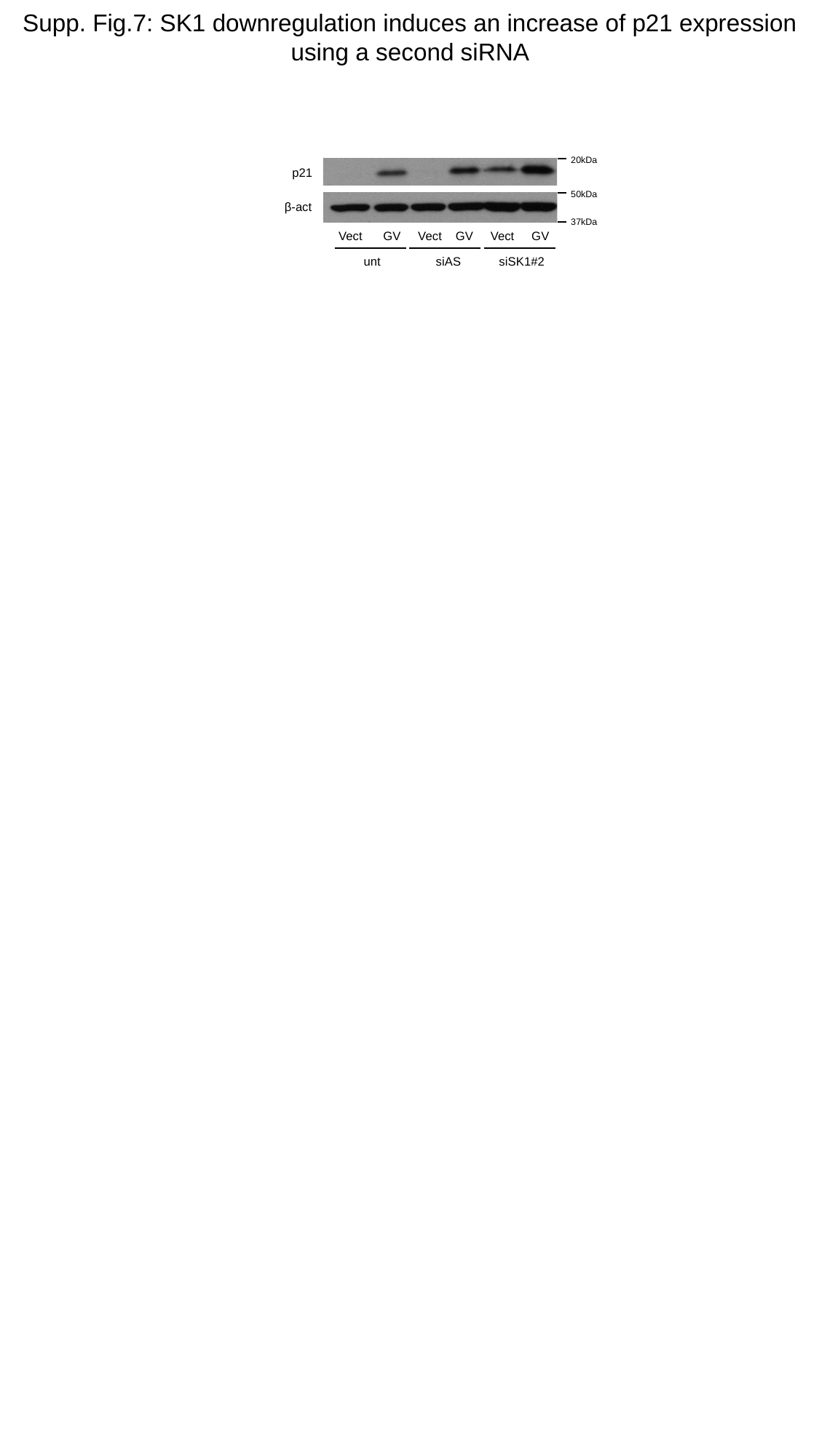

Supp. Fig.7: SK1 downregulation induces an increase of p21 expression using a second siRNA
20kDa
50kDa
37kDa
p21
β-act
Vect GV Vect GV Vect GV
unt siAS siSK1#2
