# Supplementary material for: Targeting sphingosine kinase 1 (SK1) enhances oncogene-induced senescence through ceramide synthase 2 (CerS2)-mediated generation of very-long-chain ceramides
Source: Cell Death Dis. 2021 Jan 4;12(1):27. doi: 10.1038/s41419-020-03281-4 (PMC7790826; doi:10.1038/s41419-020-03281-4)
Supplement: Supplementary file 8 — Revised Supplemental Figure 8 [file 41419_2020_3281_MOESM8_ESM.pptx]

## Slide 1
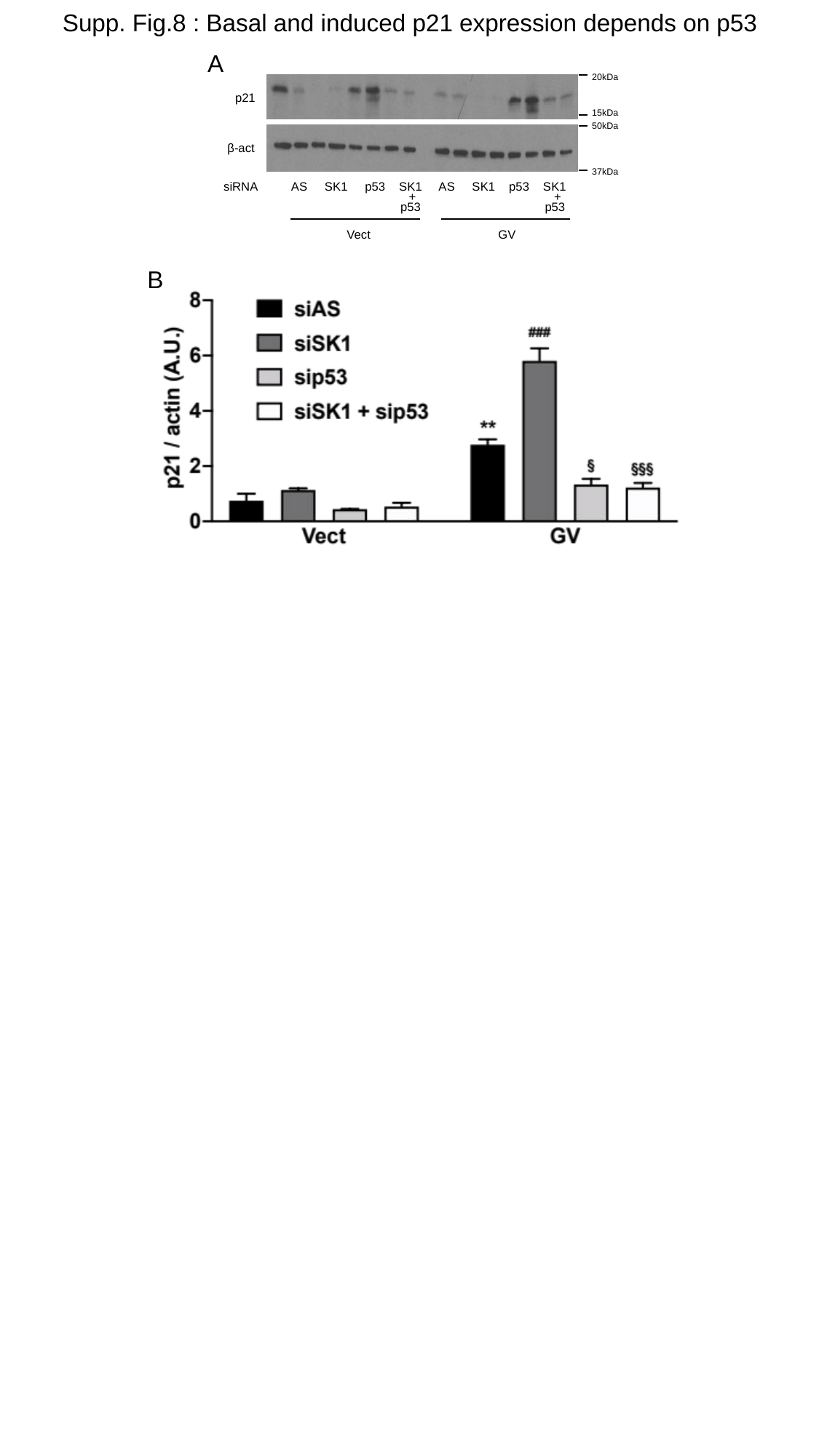

Supp. Fig.8 : Basal and induced p21 expression depends on p53
A
20kDa
15kDa
50kDa
37kDa
p21
β-act
siRNA AS SK1 p53 SK1 AS SK1 p53 SK1
+ +
 p53 p53
Vect GV
B
