# Supplementary material for: Targeting sphingosine kinase 1 (SK1) enhances oncogene-induced senescence through ceramide synthase 2 (CerS2)-mediated generation of very-long-chain ceramides
Source: Cell Death Dis. 2021 Jan 4;12(1):27. doi: 10.1038/s41419-020-03281-4 (PMC7790826; doi:10.1038/s41419-020-03281-4)
Supplement: Supplementary file 9 — Revised Supplemental Figure 9 [file 41419_2020_3281_MOESM9_ESM.pptx]

## Slide 1
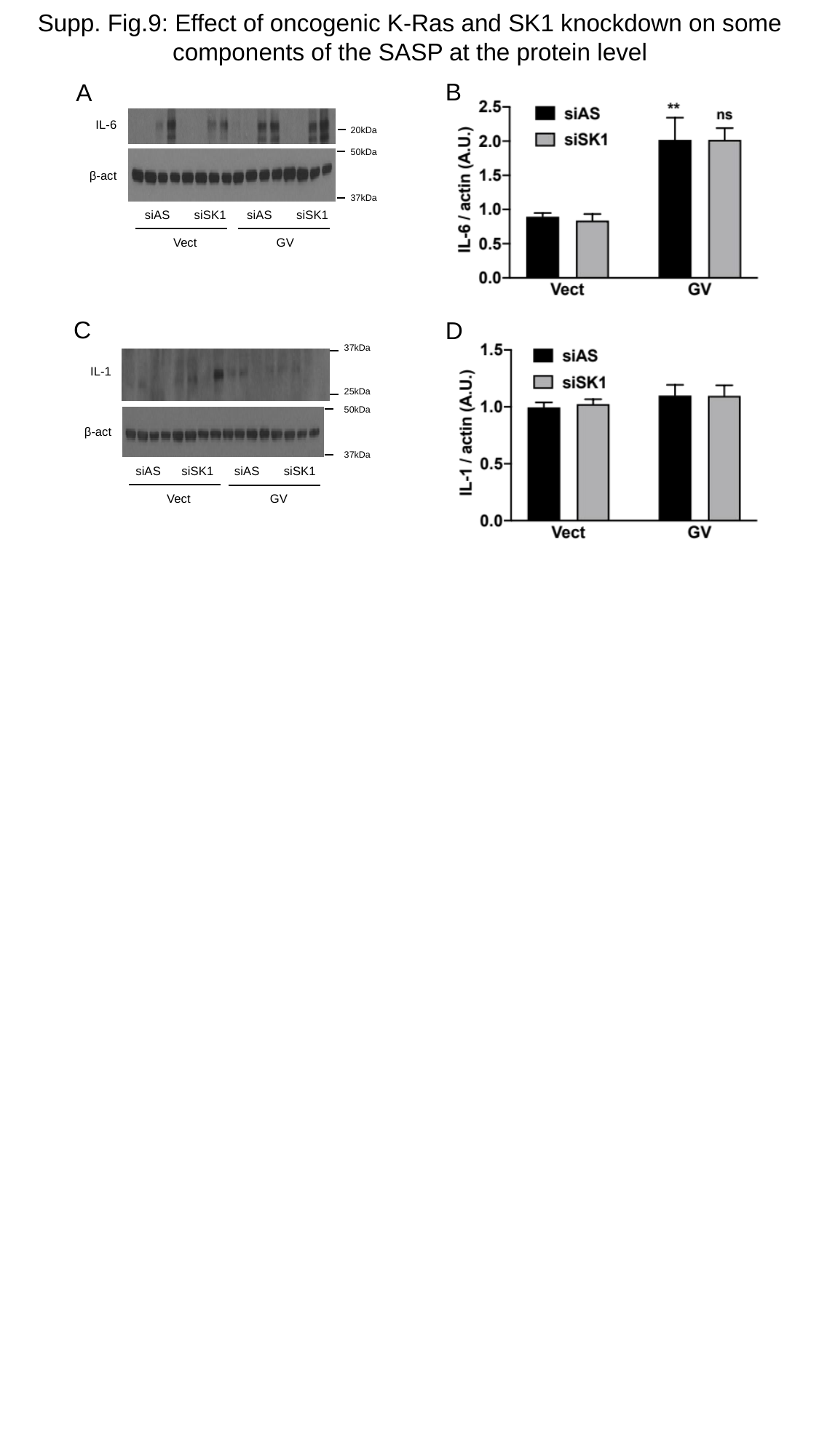

Supp. Fig.9: Effect of oncogenic K-Ras and SK1 knockdown on some components of the SASP at the protein level
B
A
IL-6
20kDa
50kDa
37kDa
β-act
siAS siSK1 siAS siSK1
Vect GV
C
D
37kDa
25kDa
50kDa
37kDa
IL-1
β-act
siAS siSK1 siAS siSK1
Vect GV
