# Supplementary material for: Targeting sphingosine kinase 1 (SK1) enhances oncogene-induced senescence through ceramide synthase 2 (CerS2)-mediated generation of very-long-chain ceramides
Source: Cell Death Dis. 2021 Jan 4;12(1):27. doi: 10.1038/s41419-020-03281-4 (PMC7790826; doi:10.1038/s41419-020-03281-4)
Supplement: Supplementary file 12 — Revised Supplemental Figure and Table legends [file 41419_2020_3281_MOESM12_ESM.docx]

**SUPPLEMENTAL FIGURE/TABLE LEGENDS**

Supp. Fig.1: Quantification of protein expression using ImageJ

A­ – B: Quantification of the western blots of Fig.1. For A, N=3, data presented as mean +/- SEM, one-way ANOVA with Dunnett’s multiple comparisons test, * means p<0,05 comparing the tested groups to the group control Vector. For B, N=5, data presented as mean +/- SEM, one-way ANOVA with Dunnett’s multiple comparisons test, * means p<0,05 comparing the tested groups to the group control Vector

C: Quantification of the western blots of Fig.2. N=5, data presented as mean +/- SEM, one-way ANOVA with Dunnett’s multiple comparisons test, * means p<0,05 comparing the tested groups to the group control Vector

D­ – E: Quantification of the western blots of Fig.3. For D, N=4, data presented as mean +/- SEM, one-way ANOVA with Dunnett’s multiple comparisons test, * means p<0,05 comparing the tested groups to the group control Vector. For E, N=3, data presented as mean +/- SEM, one-way ANOVA with Dunnett’s multiple comparisons test, * means p<0,05 comparing the tested groups to the group control Vector

F­ – G – H: Quantification of the western blots of Fig.4. For F, N=4, data are presented as mean +/- SEM, two-way ANOVA with Tukey’s multiple comparisons test, * means p<0,05 comparing the group tested to Vector DMSO, # means p<0,05 comparing the group tested to GV DMSO. For G, N=4, data are presented as mean +/- SEM, two-way ANOVA with Tukey’s multiple comparisons test, * means p<0,05 comparing the group tested to Vector control siRNA, # means p<0,05 comparing the group tested to GV control siRNA. For H, N=3, data are presented as mean +/- SEM, two-way ANOVA with Tukey’s multiple comparisons test, * means p<0,05 comparing the group tested to Vector control siRNA, # means p<0,05 comparing the group tested to GV control siRNA

I­ – J – K – L: Quantification of the western blots of Fig.8. For I, N=3, data are presented as mean +/- SEM, one-way ANOVA with Dunnett’s multiple comparisons test, * means p<0,05 comparing the group tested to the group PBS. For J, N=4, data are presented as mean +/- SEM, one-way ANOVA with Dunnett’s multiple comparisons test, * means p<0,05 comparing the group tested to the group control siRNA. For K and L, N=3, data are presented as mean +/- SEM, two-way ANOVA with Tukey’s multiple comparisons test , # means p<0,05 comparing the group tested to the group control siRNA, § means p<0,05 comparing the group SK1 siRNA to the group SK1 + FB1 or to the group SK1 + CerS2 siRNA)

Supp. Fig.2: Validation of key experimental steps in MEF

A: Observation of morphology of MEF WT or SK1-/- overexpressing Vector or GV K-Ras using bright field. Cell morphology varies across the different conditions

B: SA-ß-gal assay in MEF WT or SK1-/- overexpressing Vector or GV K-Ras. Cells were plated and then fixed and stained overnight using a SA-ß-gal staining kit. Positive cells were quantified

C: Cell growth analysis performed by cell counting using MEF WT or SK1-/- that overexpress either Vect or GV K-Ras. Number of viable cells was measured after 24 and 48h in culture

D: Colony formation assay using MEF WT or SK1-/- overexpressing Vect or GV K-Ras. Cells were plated and cultured in soft agar for 2 weeks. Number of colonies formed was quantified

Supp. Fig.3: Effect of mutant K-Ras on senescence markers and others

A – B­: Western blot analysis of p27 expression in protein extracts from cells overexpressing Vect, WT or mutant K-Ras GV or GD after 48h in culture and quantification using ImageJ. N=4, data presented as mean +/- SEM, one-way ANOVA with Dunnett’s multiple comparisons test, * means p<0,05 comparing the tested groups to the group control Vector

C – D­: Western blot analysis of p53 expression in protein extracts from cells overexpressing Vect, WT or mutant K-Ras GV or GD after 48h in culture and quantification using ImageJ. N=4, data presented as mean +/- SEM, one-way ANOVA with Dunnett’s multiple comparisons test, * means p<0,05 comparing the tested groups to the group control Vector

E – F­: Western blot analysis of Rb expression in protein extracts from cells overexpressing Vect, WT or mutant K-Ras GV or GD after 48h in culture and quantification using ImageJ. N=4, data presented as mean +/- SEM, one-way ANOVA with Dunnett’s multiple comparisons test, * means p<0,05 comparing the tested groups to the group control Vector

G – H­: Western blot analysis of Cyclin D1 expression in protein extracts from cells overexpressing Vect, WT or mutant K-Ras GV or GD after 48h in culture and quantification using ImageJ. N=4, data presented as mean +/- SEM, one-way ANOVA with Dunnett’s multiple comparisons test, * means p<0,05 comparing the tested groups to the group control Vector

I – J­: Western blot analysis of Cyclin D2 expression in protein extracts from cells overexpressing Vect, WT or mutant K-Ras GV or GD after 48h in culture and quantification using ImageJ. N=4, data presented as mean +/- SEM, one-way ANOVA with Dunnett’s multiple comparisons test, * means p<0,05 comparing the tested groups to the group control Vector

K – L­: Western blot analysis of CDK2 expression in protein extracts from cells overexpressing Vect, WT or mutant K-Ras GV or GD after 48h in culture and quantification using ImageJ. N=4, data presented as mean +/- SEM, one-way ANOVA with Dunnett’s multiple comparisons test, * means p<0,05 comparing the tested groups to the group control Vector

M – N­: Western blot analysis of CDK6 expression in protein extracts from cells overexpressing Vect, WT or mutant K-Ras GV or GD after 48h in culture and quantification using ImageJ. N=4, data presented as mean +/- SEM, one-way ANOVA with Dunnett’s multiple comparisons test, * means p<0,05 comparing the tested groups to the group control Vector

Supp. Fig.4: Lipofuscin staining to detect senescent cells

A: Replicative senescent fibroblasts were plated and kept 5 days in culture. They were then fixed and stained with Sudan Black B. Positive cells are in blue

B: Cells overexpressing Vect, WT or mutant K-Ras GV or GD were plated and kept 5 days in culture. They were then fixed and stained with Sudan Black B. Positive cells are in blue

Supp. Fig.5: Effect of PF-543, SKI-II and SK1 siRNA on SK activity

A­ – B – C: Determination of SK activity using labelling with C17-Sph. Cells were plated and treated or transfected the next day with PF-543 (100nM) or SKI-II (10μM) or control (AS) or SK1 siRNA (20nM) for 72h and then C17-Sph (250nM) was added for one hour. Cells were scraped in the lipid extraction buffer and the samples were analyzed. For A and B, N=3, data are presented as mean +/- SEM, two-way ANOVA with Sidak’s multiple comparisons test , * means p<0,05 comparing the tested groups to the group control Vector DMSO, # means p<0,05 comparing the group tested to the group GV DMSO. For C, N=5, data are presented as mean +/- SEM, two-way ANOVA with Sidak’s multiple comparisons test, * means p<0,05 comparing the tested groups to the group Vector control siRNA, # means p<0,05 comparing the group tested to the group GV control siRNA

Supp. Fig.6: Effect of SK and CerS siRNA on SK and CerS mRNA

A – B: Analysis of SK mRNA levels using RT-qPCR in cells overexpressing Vect or GV K-Ras. Cells were plated and transfected the next day with control (AS) siRNA (20nM) or directed against SK1 (2 different siRNA, siSK1#1 and siSK1#2) or SK2 and kept in culture for 72h then scraped and RNA extraction was performed on the cell pellets using a kit. Another kit was used to synthesize cDNA using 1ug of RNA. cDNA was then diluted and used as a template for RT-qPCR. The mean normalized expression was determined by using the Ct values of each gene compared to the actin expression used as a reference gene. N=2, data are presented as mean +/- SEM, no statistical analysis was performed

C: Western blot analysis of p21 expression in protein extracts from cells overexpressing Vect or mutant K-Ras GV. Cells were plated and transfected the next day with control (AS) or SK1 siRNA (siSK1#2, 20nM) for 72h

D – E – F – G – H: Analysis of CerS mRNA levels using RT-qPCR in cells overexpressing Vect or GV K-Ras. Cells were plated and transfected the next day with control (AS) siRNA (20nM) or directed against CerS1, CerS2, CerS4, CerS5 or CerS6 and kept in culture for 72h then scraped and RNA extraction was performed on the cell pellets using a kit. Another kit was used to synthesize cDNA using 1ug of RNA. cDNA was then diluted and used as a template for RT-qPCR. The mean normalized expression was determined by using the Ct values of each gene compared to the actin expression used as a reference gene. N=2, data are presented as mean +/- SEM, no statistical analysis was performed

Supp. Fig.7: SK1 downregulation induces an increase of p21 expression using a second siRNA

Western blot analysis of p21 expression in cell lysates from cells overexpressing Vect or GV K-Ras. Cells were plated and transfected the next day with siRNA control (AS) or SK1#2 (20nM) for 72h. Cells were then lysed and protein level of p21 was determined

Supp. Fig.8: Basal and induced p21 expression depends on p53

A – B:­ Western blot analysis of p21 expression in protein extracts from cells overexpressing Vect or K-Ras GV. Cells were plated and transfected the next day with siRNA control (AS) or SK1 siRNA or p53 siRNA or both SK1 and p53 siRNA (20nM) for 72h. Cells were then lysed and protein level of p21 was determined and quantified using ImageJ. N=3, data are presented as mean +/- SEM, two-way ANOVA with Tukey’s multiple comparisons test , * means p<0,05 comparing the group Vect control siRNA to the group GV control siRNA, # means p<0,05 comparing the group GV control siRNA to the group GV SK1 siRNA, § means p<0,05 comparing the group GV SK1 siRNA to the group SK1 + p53 siRNA

Supp. Fig.9: Effect of oncogenic K-Ras and SK1 knockdown on some components of the SASP at the protein level

A – B­: Western blot analysis of IL-6 expression in protein extracts from cells overexpressing Vect or mutant K-Ras GV. Cells were plated and transfected the next day with siRNA control (AS) or SK1 (20nM) for 72h. Cells were then lysed and protein level of IL-6 was determined and quantified using ImageJ. N=4, data are presented as mean +/- SEM, two-way ANOVA with Tukey’s multiple comparisons test, * means p<0,05 comparing the group Vect control siRNA to the group GV control siRNA, # means p<0,05 comparing the group GV control siRNA to the group GV SK1 siRNA

C – D­: Western blot analysis of IL-1 expression in protein extracts from cells overexpressing Vect or mutant K-Ras GV. Cells were plated and transfected the next day with siRNA control (AS) or SK1 (20nM) for 72h. Cells were then lysed and protein level of IL-1 was determined and quantified using ImageJ. N=4, data are presented as mean +/- SEM, two-way ANOVA with Tukey’s multiple comparisons test, * means p<0,05 comparing the group Vect control siRNA to the group GV control siRNA, # means p<0,05 comparing the group GV control siRNA to the group GV SK1 siRNA

Supp. Fig.10: Relative expression of CerS mRNA

Analysis of CerS mRNA levels using RT-qPCR in cells overexpressing Vect or GV K-Ras. Cells were plated and kept in culture for 48h then scraped and RNA extraction was performed on the cell pellets using a kit. Another kit was used to synthesize cDNA using 1ug of RNA. cDNA was then diluted and used as a template for RT-qPCR. All CerS and Actin TaqMan probes were used. The mean normalized expression was determined by using the Ct values. N=5, data are presented as mean +/- SEM, no statistical analysis was performed

Supp. Table 1: List of siRNA, antibodies and TaqMan assays
